# Stabilizing Stretchable Organic Transistors Through Small‐Molecule Additive Blending for Ultra‐Sensitive Pesticide Detection

**DOI:** 10.1002/advs.202513397

**Published:** 2025-10-13

**Authors:** Weiyu Wang, Liya Dai, Xiangxiang Li, Xiaoying Zhang, Huiqi Yang, Xinran Zheng, Yan Wang, Xin Ye, Hui Yang, Wenping Hu

**Affiliations:** ^1^ State Key Laboratory of Advanced Materials for Intelligent Sensing Key Laboratory of Organic Integrated Circuit Ministry of Education & Tianjin Key Laboratory of Molecular Optoelectronic Sciences Department of Chemistry School of Science & Institute of Molecular Aggregation Science Tianjin University Tianjin 300072 China

**Keywords:** extended‐gate, high mobility, organic field‐effect transistors, pesticide sensors, stretchable transistors

## Abstract

Intrinsically stretchable organic field‐effect transistors (OFETs) have emerged as a promising platform for real‐time, wearable sensing. However, achieving high charge carrier mobility while ensuring long‐term stability under extreme deformation remains a critical challenge. Herein, a stabilization strategy employing 2,4,5,7‐tetranitrofluorenone (TeNF) as a water‐displacing additive in indacenodithiophene‐co‐benzothiadiazole (IDTBT) polymer semiconductor is demonstrated. Thermodynamically favored TeNF‐polymer interactions selectively exclude ambient moisture from nanoscale voids, achieving dual enhancement in mobility (3.50 cm^2^ Vs^−1^) and environmental stability. The fully stretchable transistors maintain ideal electrical characteristics under 100% strain and after 60 days of ambient storage. Furthermore, by integrating an extended‐gate configuration, highly sensitive and precise detection of chlorpyrifos pesticide is achieved, reaching a detection limit as low as 0.032 ppb and a fast response time of 6.4 s toward 1 ppb chlorpyrifos. These results pave the way for next‐generation stretchable organic transistors with enhanced reliability for advanced sensing applications.

## Graphical abstract

This study introduces a small‐molecule additive to improve the stability of stretchable organic field‐effect transistor (OFET). Furthermore, stretchable OFET‐based pesticide sensors with extended‐gate electrodes are constructed. It achieves highly sensitive and precise detection of chlorpyrifos pesticide, reaching a detection limit as low as 0.032 ppb. The innovation offers high stability and accuracy for next‐generation sensing applications.

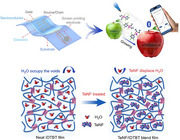

## 1 Introduction

Stretchable organic field‐effect transistors (OFETs) offer unique advantages, including intrinsic conformal adhesion, versatile device architectures, multiple sensing mechanisms, and inherent signal amplification.^[^ ^1^, ^2^, ^3^, ^4^, ^5^ ^]^ Under mechanical deformation, these devices maintain precise real‐time monitoring of hazardous gases,^[^ ^6^ ^]^ human biochemical markers,^[^ ^7^, ^8^, ^9^ ^]^ and electrophysiological signals.^[^ ^10^, ^11^, ^12^ ^]^ Recent advancements have demonstrated their utility in healthcare (e.g., on‐skin glucose sensing^[^ ^9^ ^]^ and cardiac rhythm monitoring^[^ ^13^ ^]^) and augmented reality systems (e.g., electrooculographic control^[^ ^11^ ^]^). Notably, stretchable electronics demonstrate growing potential in smart agriculture through non‐invasive plant adhesion, facilitating continuous microenvironment and physiological tracking across growth cycles.^[^ ^14^, ^15^, ^16^ ^]^ Their multifunctional capabilities enable OFETs to serve critical roles in advancing bioelectronic healthcare technologies and agricultural monitoring technologies.

The stability of intrinsically stretchable OFETs is crucial for sustained and reliable sensing applications, requiring both operational stability and long‐term storage stability.^[^ ^17^, ^18^, ^19^, ^20^ ^]^ Water‐induced degradation mechanisms, particularly through hygroscopic charge trapping at polymer‐semiconductor interfaces, critically compromise long‐term operational reliability.^[^ ^21^, ^22^, ^23^ ^]^ While conventional encapsulation methods using rigid polymers (e.g., poly(methyl methacrylate), polyimide) mitigate moisture intrusion, their mechanical mismatch with stretchable substrates induces interfacial delamination under strain.^[^ ^24^, ^25^ ^]^ Dopant stabilization approaches have been explored to enhance device stability, yet these often result in undesirable increases in off‐state currents.^[^ ^26^, ^27^, ^28^ ^]^ Thus, achieving stable, high‐performance stretchable OFETs remains a fundamental challenge and a critical opportunity for advancing next‐generation sensing technologies.

**Figure 1:**
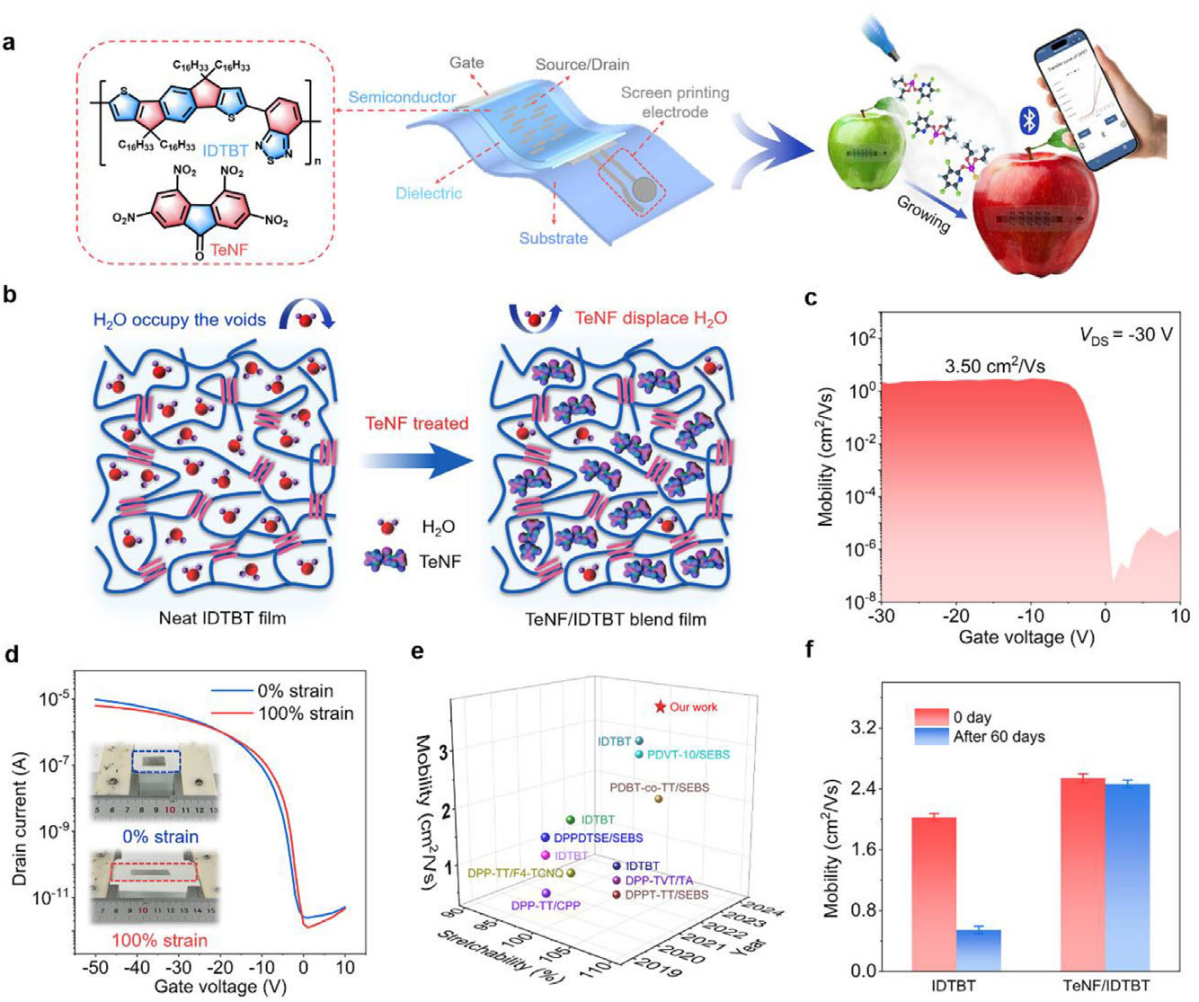
High‐performance stretchable pesticide sensor based on TeNF/IDTBT blend transistor. a) Schematic representation of a pesticide sensor with an intrinsically stretchable transistor. b) Illustration of the stabilization mechanism, highlighting water displacement by TeNF within the polymer semiconductor. c) Gate‐dependent saturation mobility curves of stretchable OFET with 3 mol% TeNF/IDTBT blend film. d) Transfer curves and photographs of the fully stretchable OFET under 0% and 100% strain. e) Comparison of previously reported stretchable transistors with our high‐performance transistor in mobility and stretchability. f) Influence of TeNF on storage stability performance of pristine IDTBT OFET.

In this work, we develop a small‐molecule additive blending strategy to enhance the stability of stretchable OFETs (**Figure** **1**a). The co‐blending of indacenodithiophene‐co‐benzothiadiazole (IDTBT) with 2,4,5,7‐tetranitrofluorenone (TeNF) effectively displaces water molecules from the nanoscale voids within the polymer semiconductor through competitive molecular interactions (Figure 1b). The fully stretchable OFETs fabricated with TeNF/IDTBT blend films achieved a high mobility of 3.50 cm^2^ Vs^−1^, which remains stable under up to 100% tensile strain (Figure 1c,d). Our results represent both the highest recorded mobility and the largest strain tolerance among IDTBT‐based OFETs (Figure 1e; Table S1, Supporting Information). Furthermore, the devices preserved ideal electrical performance even after 60 days of ambient storage (Figure 1f). Integrating these stretchable OFETs with extended‐gate electrodes enables the fabrication of highly adhesive and conformal pesticide sensors capable of detecting chlorpyrifos with remarkable sensitivity and selectivity, achieving a detection limit as low as 0.032 ppb. These findings highlight the significant potential of our blending strategy in advancing stretchable OFET‐based sensing technologies, offering high stability and accuracy for next‐generation sensing applications.

## 2 Results and Discussion

### 2.1 Fabrication and Characterization of TeNF/IDTBT Blend Semiconductor Films

**Figure 2:**
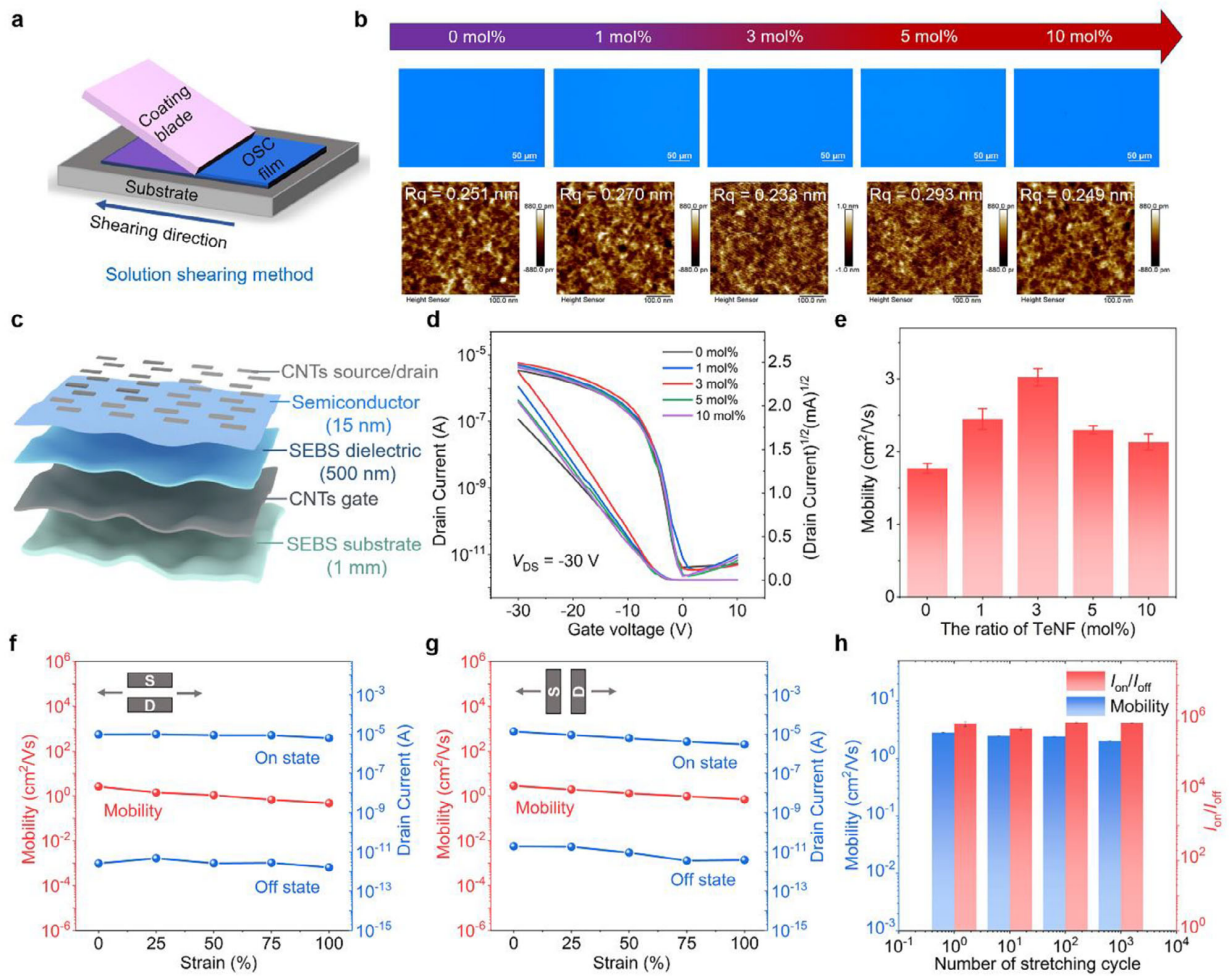
Intrinsically stretchable transistors fabricated from TeNF blend optimized semiconductor films. a) Schematic illustration of the solution shearing method used to fabricate organic semiconductor films on an OTS‐treated Si/SiO_2_ substrate. b) OM and AFM images of semiconductor films with varying TeNF concentrations (0, 1, 3, 5, 10 mol%). c) Device structure of a bottom‐gate top‐contact stretchable transistor with TeNF/IDTBT as the semiconductor, CNTs as the gate and source/drain electrodes (channel length: 100 µm, channel width: 400 µm), SEBS H1052 (500 nm thick) as the dielectric, and SEBS H1062 (1 mm thick) as the stretchable substrate. d,e) Transfer curves d) and mobilities e) of stretchable OFETs with varying TeNF concentrations. f,g) Changes in the drain current and mobility (calculated with device geometry and dielectric capacitance under strain, as detailed in Table S2, Supporting Information) when strain is parallel f) and perpendicular g) to the charge transport direction. h) *I*_on/off_ and mobility during 1000 repeated stretching cycles at 30% strain.

In this study, the polymer IDTBT was selected as the host polymer semiconductor, while the nitrofluorenone derivative TeNF served as the small‐molecule additive. The high solubility of both components in chlorobenzene enabled homogeneous blending. The semiconductor films were fabricated via precisely controlled solution shearing on octadecyl trichlorosilane (OTS)‐modified Si/SiO_2_ substrates (**Figure** **2**a). To achieve a hybrid polymer semiconductor with optimal morphology and electric properties, the preparation conditions were optimized by adjusting the blending ratio of TeNF and IDTBT from 0 to 10 mol%, with a solution concentration of 5 mg mL^−1^, a shearing speed of 0.52 mm s^−1^, and an annealing temperature of 150 °C. The presence of the −NO_2_ peak in the X‐ray photoelectron spectroscopy (XPS) of the TeNF/IDTBT blend film confirms the successful incorporation of the TeNF additive (Figure S1, Supporting Information). The optical microscopic (OM) images of the semiconductor films, exhibiting a uniform color, provide clear evidence of their homogeneity. Surface roughness, measured by atomic force microscopy (AFM), was determined to be 0.251 nm for the IDTBT film and 0.233 nm for the 3 mol% TeNF/IDTBT film (Figure 2b). Additionally, the roughness of the SEBS dielectric layer was measured to be 0.3 nm (Figure S2, Supporting Information). Thus, the incorporation of TeNF resulted in smooth, uniform TeNF/IDTBT blend films, which optimize charge transport and promote the formation of high‐quality contact interfaces, thereby enhancing device performance.

### 2.2 Electrical Characterization of Stable Stretchable OFETs

The fully intrinsically stretchable OFETs based on TeNF/IDTBT blend films were fabricated with a bottom‐gate top‐contact structure with CNTs as the gate, source/drain electrodes, and SEBS as the dielectric layer and substrate (Figure 2c). The overall fabrication flow of highly integrated intrinsically stretchable transistor arrays is provided in Figure S3 (Supporting Information). Figure 2d presents the transfer characteristics of stretchable OFETs incorporating TeNF‐treated films with varying additive concentrations, ranging from 0% (neat IDTBT) to 10 mol%. The corresponding mobilities of the devices, extracted from Figure 2d, are shown in Figure 2e. The fully stretchable OFET achieved a peak mobility of 3.50 cm^2^ Vs^−1^ at a TeNF concentration of 3 mol%, compared to only 1.55 cm^2^ Vs^−1^ for the pristine IDTBT‐based OFET. Additionally, we evaluated the statistical distribution of mobility across 16 independently fabricated 3 mol% TeNF/IDTBT devices, revealing an average mobility of 3.30 cm^2^ Vs^−1^. The mobility of the 16 devices showed a relative standard deviation (RSD) of 3.89%, indicating excellent device‐to‐device uniformity in mobility (Figure S4, Supporting Information). Moreover, the 3 mol% TeNF/IDTBT blend OFET exhibited a typical ohmic‐contact output curve (Figure S5, Supporting Information), demonstrating nearly ideal transistor characteristics.^[^ ^29^ ^]^ Since 3 mol% blend films exhibited optimal mobility, we chose this concentration to further investigate the device's mechanical stretchability and stability. Mechanical stretchability was examined by monitoring the film morphology and transistor performance under tensile strain applied both parallel and perpendicular to the channel direction. Optical microscope images confirmed that films with or without TeNF remained free of cracks under varying tensile strains (Figure S6, Supporting Information). The transfer characteristics exhibited negligible degradation performance even under 100% strain, regardless of whether stretching occurred parallel or perpendicular to the channel direction (Figure 2f,g; Figure S7, Supporting Information). The drain current and estimated mobility mostly retained the initial value. Furthermore, the fully stretchable OFET demonstrated good robustness, maintaining stable performance after 1000 repeated stretching cycles to 30% strain (Figure 2h; Figure S8, Supporting Information). Cyclic stretching tests were performed on 10 OFET devices. 10 devices retained over 52.3% of their original mobility after 1000 cyclic stretching cycles to 30% strain. The statistical analysis of their performance before and after stretching revealed excellent cyclic stability, demonstrating excellent device‐to‐device uniformity in strain tolerance (Figure S9, Supporting Information). These results indicate the superior stretchability of the devices and confirm that the incorporation of TeNF does not compromise the intrinsic stretchability of the IDTBT semiconductor film.

### 2.3 Stability of Stretchable Devices

**Figure 3:**
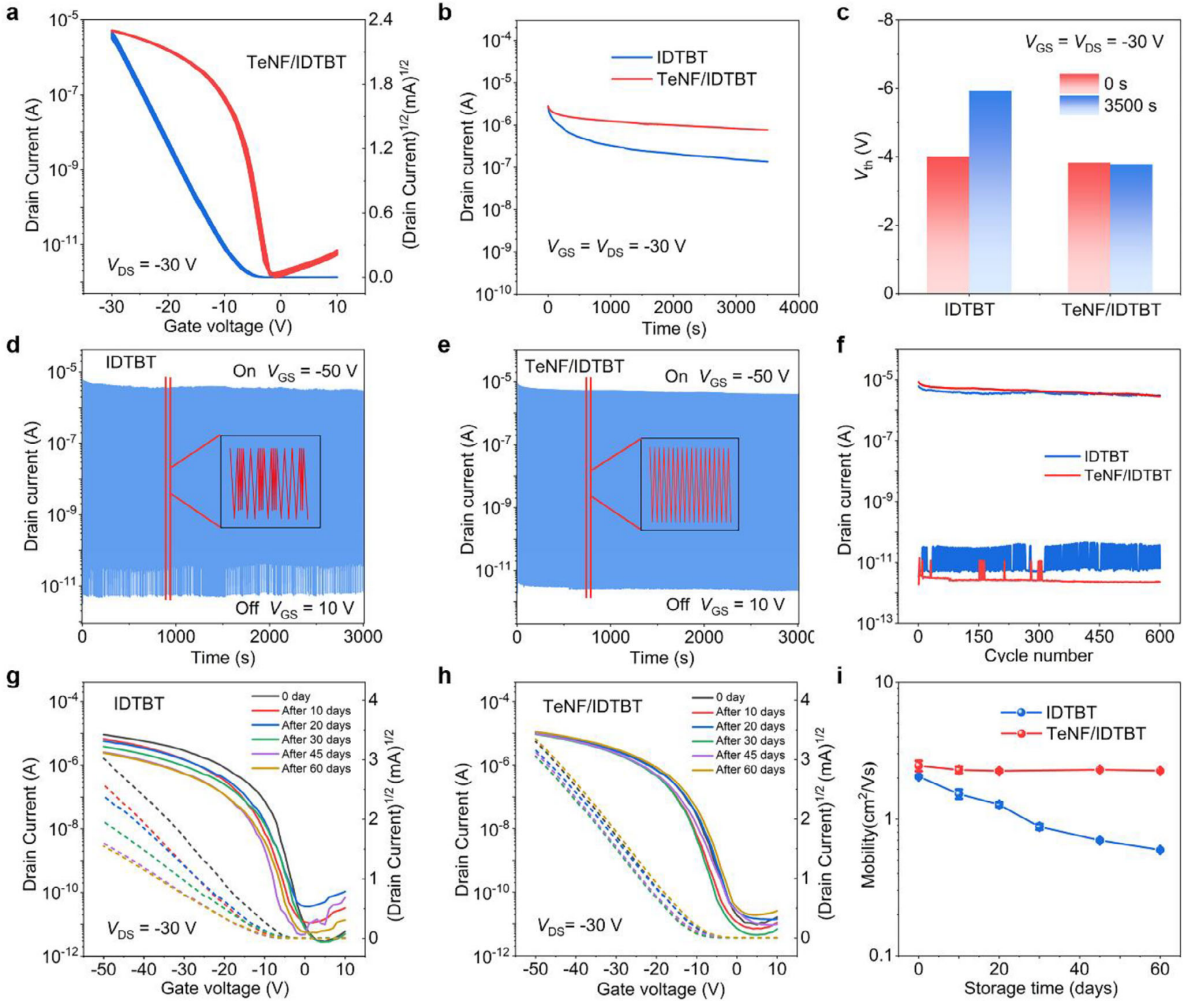
Operational and long‐term storage stability in air. a) Transfer curves from 50 sweeping cycles for a single stretchable transistor with 3 mol% TeNF/IDTBT blend film. b,c) Bias stressing stability b), and threshold voltage drift c) of pristine IDTBT OFET and TeNF/IDTBT blend OFET (*V*_DS_ = *V*_GS_ = −30 V). d,e) Cyclic stability of OFET with neat IDTBT film d) and TeNF/IDTBT blend film e) during 3000 s continuous test (test frequency = 10 Hz, *V*_DS_ = −30 V, *V*_GS_ = −30 and 0 V, alternately). f) Switching current change of pristine IDTBT device (blue line) and TeNF/IDTBT blend device (red line) over 600 switching cycles. g,h) Long‐term storage stability of pristine IDTBT OFET g) and TeNF/IDTBT blend OFET h) exposed to air. i) Extracted the mobilities of both devices over varying storage times.

To assess the reliability of our devices in long‐term real‐time sensing, both operational stability and long‐term stability were evaluated. We performed cyclic sweeping tests on the TeNF/IDTBT blend OFET, and the transfer curves of the device exhibited no appreciable changes after 50 sweeping cycles. The device retained a mobility of 3.48 cm^2^ Vs^−1^ even after 50 cycle scans (**Figure** **3**a). The on‐state current of the device does not degrade significantly, and could maintain 10^6^ A, indicating its desirable field‐effect characteristics. We further performed bias stress measurements in air. After stressing the devices at a constant high bias of *V*_DS_  =  *V*_GS_  =  −30 V, the TeNF/IDTBT blend device exhibited significantly slower drain current degradation over 3500 s, compared to the pristine IDTBT device (Figure 3b). Moreover, threshold voltage (*V*_th_) measured before and after the bias stress period revealed a significantly reduced *V*_th_ drift (0.05 V) in the TeNF/IDTBT blend device, while the *V*_th_ drift of transistors with pristine IDTBT films was 1.93 V, highlighting their enhanced bias stress stability (Figure 3c). Furthermore, we applied the gate voltage of *V*_GS_ = −50 V and *V*_GS_ = 0 V to establish the pristine IDTBT device and TeNF/IDTBT blend device in on and off states, as shown in Figure 3d,e. After 600 cycles of stress testing, the TeNF/IDTBT blend device maintained stable switching performance with negligible current attenuation, while the pristine IDTBT device exhibited an unstable current switching ratio. The on‐state current and off‐state current extracted from tests are presented in Figure 3f. The *I*_on/off_ of TeNF/IDTBT blend OFET exceeds 10^6^, which can meet the needs of practical sensing applications. In addition to operational stability, the long‐term stability was also assessed by exposing both device types to ambient air (humidity range of 60–80%) for 60 days without encapsulation. The transfer characteristics confirm the excellent long‐term stability of the TeNF/IDTBT blend devices. The pristine IDTBT OFET showed a 71% degradation in mobility over this period (Figure 3g), whereas TeNF/IDTBT blend OFET exhibited minimal change, maintaining a high mobility of 2.25 cm^2^ Vs^−1^ even after 60 days (Figure 3h,i). The *I*_on/off_ of pristine IDTBT and TeNF/IDTBT blend device remains at 73.7% and 2.6% of the initial value, respectively. These results confirmed that the effectiveness of our small‐molecule additive blending strategy, the incorporation of TeNF, significantly enhanced the operational stability and long‐term stability of the devices, rendering them highly promising for advanced real‐time sensing applications.

### 2.4 Mechanisms of Mobility and Stability Enhancement

The performance enhancement mechanisms were systematically investigated through complementary spectroscopic, electrical, and computational analysis. Interactions between TeNF and IDTBT were analyzed using electron paramagnetic resonance (EPR) and UV–Vis–NIR absorption spectroscopy (Figure S10, Supporting Information). The absence of EPR signals and spectral shifts in TeNF/IDTBT blend films revealed no charge transfer complex formation, explaining the stable off‐state current across TeNF concentrations.^[^ ^27^ ^]^ Raman analysis demonstrated enhanced polymer coplanarity through the *I*_1536_/*I*_1612_ ratio increasing from 2.54 (pristine IDTBT film) to 2.64 (3 mol% TeNF/IDTBT film) (Figure S11, Supporting Information). This structural optimization reduces π‐conjugation defects, facilitates carrier transport along the polymer backbone, and contributes to enhanced mobility.^[^ ^30^, ^31^ ^]^

**Figure 4:**
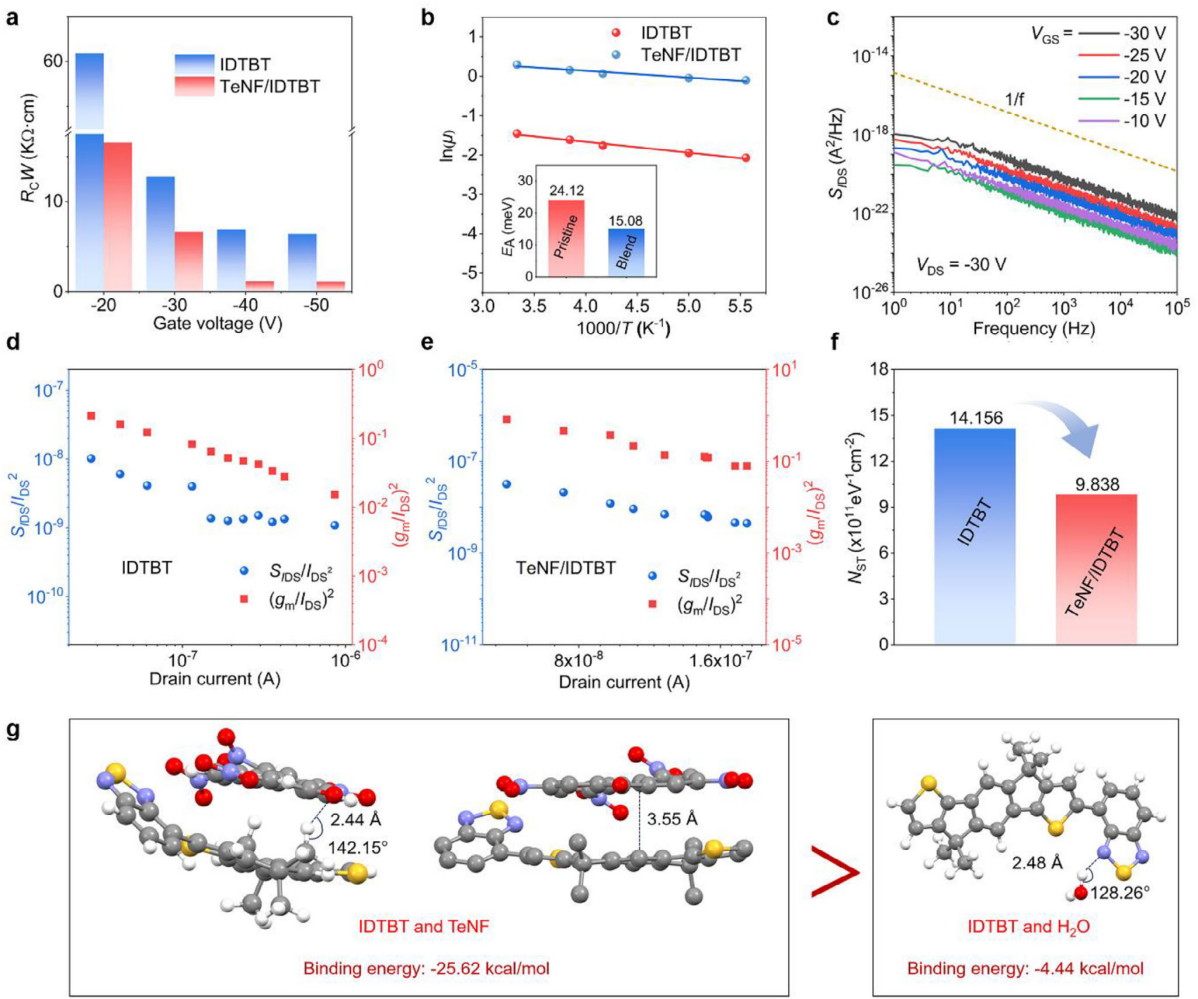
Mechanisms of mobility and stability enhancement. a,b) Contact resistance a) and activation energy b) measured for fully stretchable transistors with and without TeNF. c) Typical drain current PSD of 3 mol% TeNF/IDTBT blend OFET as a function of the frequency for different gate voltages. d,e) Normalized *S_I_*_DS_/*I*_DS_^2^ at a fixed frequency of 20 Hz, plotted versus *I*_DS_ of pristine IDTBT device d) and TeNF/IDTBT blend device e), where *g*_m_ was transconductance. f) Extracted surface trap density (*N*_ST_) measured by LFN. g) Computational evaluation of the interactions between the additive and polymer backbone, the water and polymer backbone, and the additive and water based on DFT.

Electrical characterization revealed critical interfacial improvements induced by TeNF. Contact resistance (*R*_c_) was quantified using the transfer line method across the gate voltage range of *V*_GS_ =  −50 to −20  V (**Figure** **4**a; Figure S12, Supporting Information). The TeNF/IDTBT blend OFET exhibited significantly reduced *R*_c_ compared to the pristine IDTBT OFET. This interfacial improvement resulted in significantly higher mobility.^[^ ^27^, ^32^ ^]^ The activation energy (*E*_A_) for charge transport in semiconductor films dropped from 24.12 to 15.08 meV (Figure 4b; Figure S13, Supporting Information), indicating shallow trap mitigation after the introduction of TeNF.^[^ ^33^ ^]^ Furthermore, the grazing‐incidence small‐angle X‐ray scattering (GISAXS) patterns showed that the incorporation of the additive TeNF enhanced the intensity of the (100) and (010) diffraction peaks in the semiconductor film (out‐of‐plane). This enhancement in peak intensity implies improved crystallinity and molecular packing, signifying a reduction in trap states at the semiconductor‐dielectric interface (Figure S14, Supporting Information). Meanwhile, the interface trap density (*N*_ST_) was calculated from the measured subthreshold swing (*SS*) to quantify the quality of the semiconductor‐dielectric interface. The results indicated that the incorporation of TeNF reduced the interface trap density from 3.133 × 10^11^ eV^−1^ cm^−2^ to 1.840 × 10^11^ eV^−1^ cm^−2^ (Figure S15, Supporting Information). Low‐frequency noise (LFN) analysis verified interfacial trap density reduction from 1.4156 × 10^12^ eV^−1^ cm^−2^ in pristine IDTBT OFET to 9.838 × 10^11^ eV^−1^ cm^−2^ in TeNF/IDTBT blend OFET through 1/*f*‐parameterized power spectral density (PSD) analysis at 20 Hz (Figure 4c‐˗f; Figure S16, Supporting Information). All the results confirmed that TeNF effectively mitigates trap state density at the interface, enhances carrier mobility, and suppresses degradation pathways critical for operational stability.

The stabilization mechanism was further elucidated through density functional theory (DFT) calculations at the WB97XD/6‐31G(d,p) level (Figure 4g). In the TeNF/IDTBT system, dual non‐covalent interactions were identified: a hydrogen bond (C─H···O distance of 2.44 Å) and a Van der Waals interaction (the distance between the centroids of conjugate planes of 3.55 Å). By contrast, the competing H_2_O/IDTBT interaction exhibited a shorter hydrogen bond distance (O─H···N distance of 2.18 Å) but significantly weaker binding energy (−4.44 kcal mol^−1^ vs TeNF/IDTBT: −25.62 kcal mol^−1^). The bonding energy is −6.60 kcal mol^−1^ in the TeNF/H_2_O system, which is significantly weaker than TeNF/IDTBT (Figure S17, Supporting Information). Consequently, the introduction of the additive TeNF suppresses the H_2_O/IDTBT interaction, displaces the water within the polymer nanometre‐sized voids, and passivates the trap states. Meanwhile, the influence of the other two additives (tetramethylammonium iodide (NMe_4_I), bis(pentafluorophenyl)zinc [Zn(C_6_F_5_)_2_]) on the storage stability of the device was compared (Figure S18, Supporting Information). The performance of NMe_4_I/IDTBT and Zn(C_6_F_5_)_2_/IDTBT OFETs exhibited significant degradation after 60 days, which revealed that ionic and Lewis acid additives are ineffective at enhancing OFET device stability. By contrast, the introduction of TeNF prevents water molecules from accessing the polymer backbone and occupying the free volume in the film, which suppresses the formation of hydrogen bonds between IDTBT and H_2_O. Also, the incorporation of the TeNF additive also reduced the contact resistance, activation energy, and interface trap density, thereby successfully improving the electrical performance and stability of the device.

### 2.5 Applicability of TeNF Stabilization Strategy

**Figure 5:**
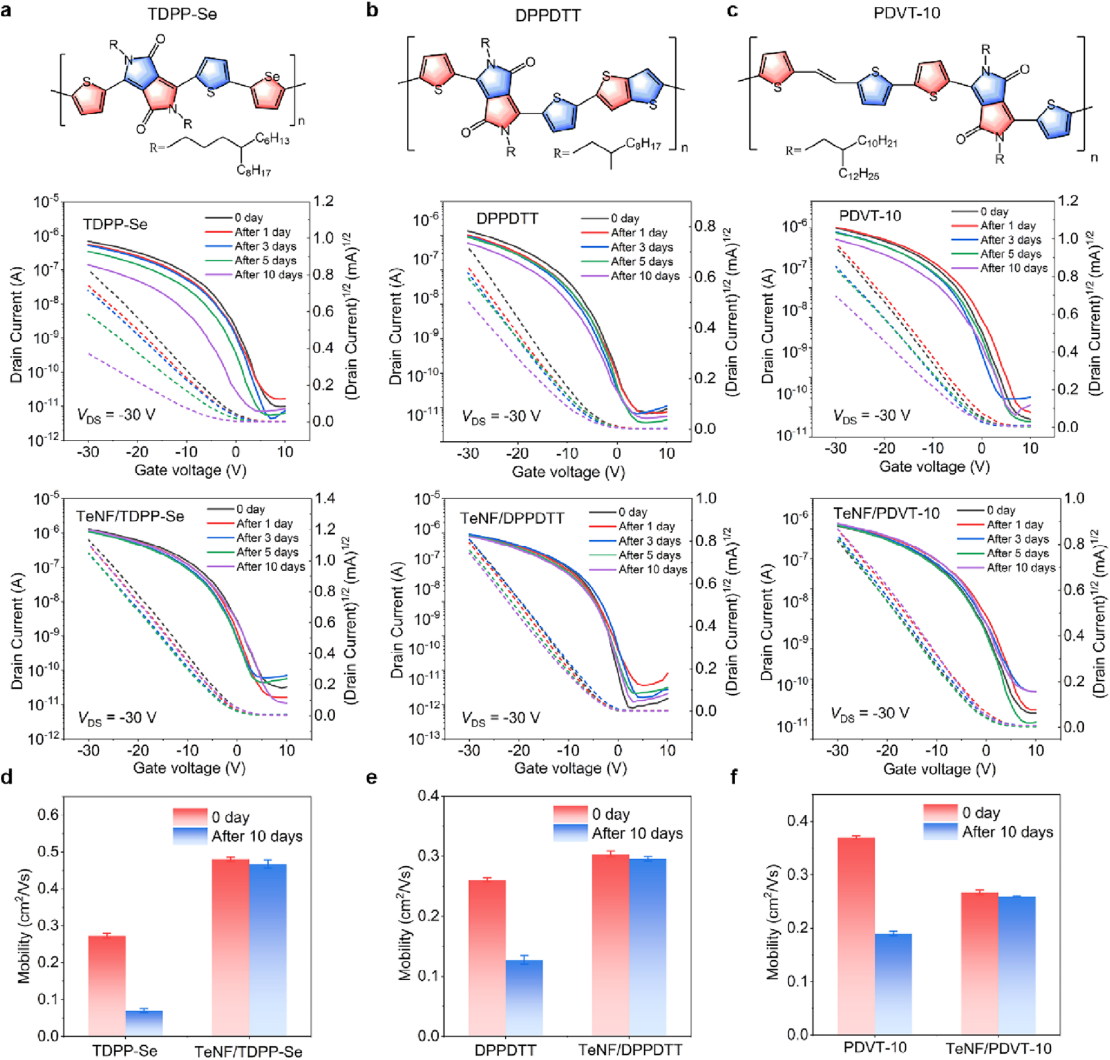
Applying the small‐molecule additive blending strategy to three distinct conjugated polymers to improve their stability. a–c) Transfer curves of neat OFETs devices (middle) and blend OFETs devices (below), for conjugated polymers of TDPP‐Se a), DPPDTT b), and PDVT‐10 c), with the chemical structures shown above. d–f) Influence of TeNF on storage stability performance of pristine TDPP‐Se OFET d), DPPDTT OFET e), and PDVT‐10 OFET f).

To examine the versatility of our small‐molecule additive blending strategy, we applied it to three other high‐performance conjugated polymers, with their chemical structures shown in **Figure** **5**a‐˗c. All devices based on neat conjugated polymer films severely degraded in mobility when stored in the ambient environment for 10 days. After the introduction of small‐molecule TeNF, the devices based on blend polymer films showed high stability enhancement and no obvious degradation after 10 days. For example, the pristine TDPP‐Se, DPPDTT, and PDVT‐10 OFET showed a 72.0, 52.7, and 47.2% degradation in mobility over this period, whereas TeNF/TDPP‐Se, TeNF/DPPDTT, and TeNF/PDVT‐10 blend OFET exhibited minimal change, maintaining the initial mobility of 0.47, 0.29, and 0.26 cm^2^ Vs^−1^ even after 10 days, respectively (Figure 5d–f; Figure S19, Supporting Information). Similarly, we employed DFT to calculate the binding energies between polymer semiconductors (TDPP‐Se, DPPDTT, PDVT‐10) and the additive (TeNF), as well as between each polymer and water. The results revealed that the polymer‐additive interactions were significantly stronger than the polymer‐water interactions in all cases (TeNF/TDPP‐Se: −22.72 kcal mol^−1^ vs H_2_O/TDPP‐Se:−5.91 kcal mol^−1^; TeNF/DPPDTT:−26.07 kcal mol^−1^ vs H_2_O/DPPDTT: −5.85 kcal mol^−1^; TeNF/PDVT‐10: −20.49 kcal mol^−1^ vs H_2_O/PDVT‐10: −5.99 kcal mol^−1^) (Figure S20, Supporting Information). Consequently, the additive preferentially interacts with the polymer, fills the nanoscale voids within its microstructure, and displaces the trapped water molecules. These results demonstrate that the additive TeNF effectively removes water and passivates water‐induced traps in polymer semiconductor films, significantly enhancing the operational stability of OFETs. Our findings establish the molecular additives as an effective strategy for enhancing the operational and long‐term storage stability of polymer‐based OFET devices.

### 2.6 Ultrasensitive Chlorpyrifos Pesticide Sensors Based on the Stretchable OFETs

**Figure 6:**
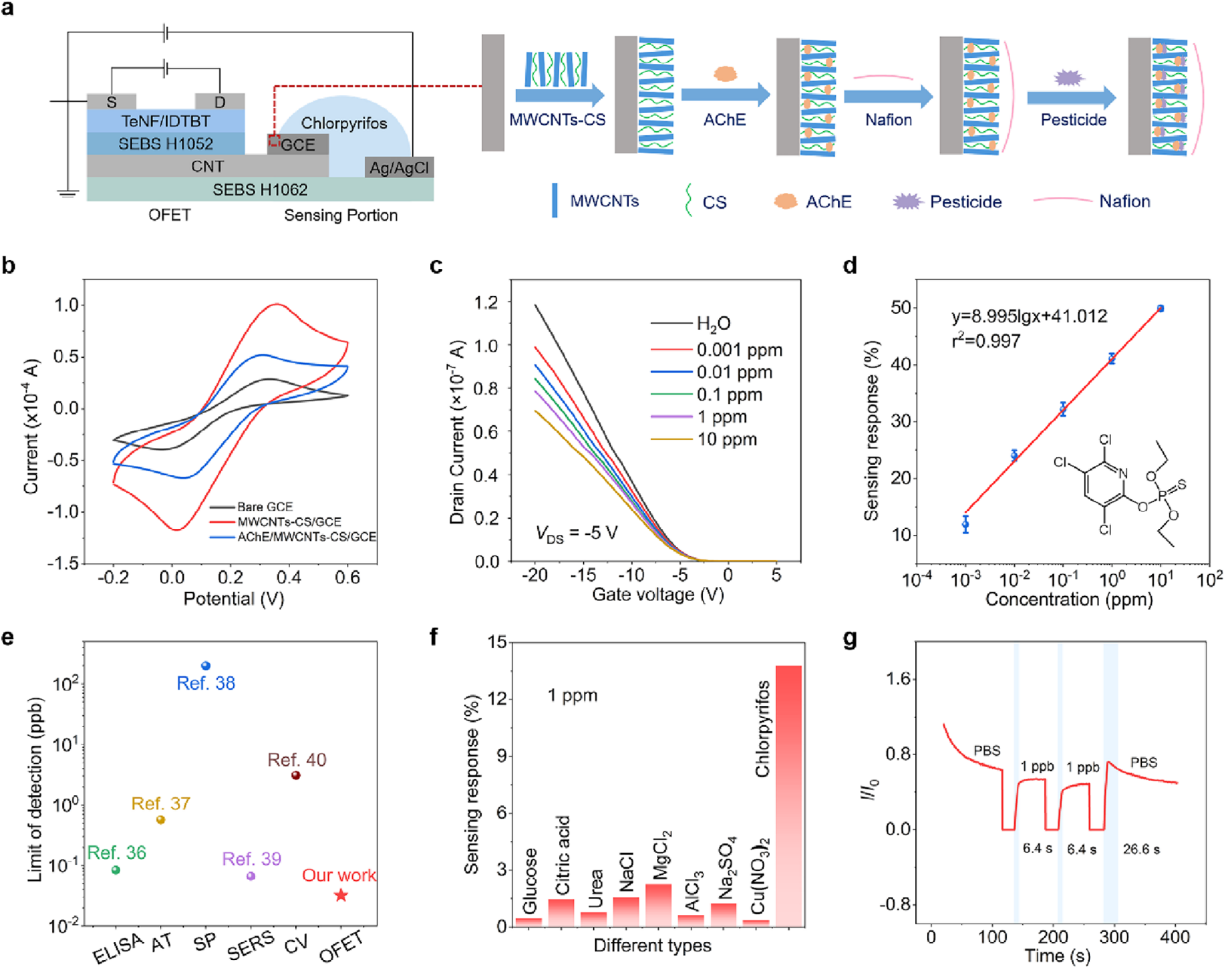
High‐sensitivity pesticide sensing based on an extended‐gate organic transistor sensor. a) Schematic of a pesticide sensor based on intrinsically stretchable OFET and the layer‐by‐layer modification process of the extended‐gate electrode. b) Cyclic voltammetry of bare GCE, MWCNTs‐CS/GCE, and AchE/MWCNTs‐CS/GCE. c) Transfer curves of the OFET‐based sensor under varying concentrations of chlorpyrifos. d) Sensing response of the OFET sensor to chlorpyrifos concentrations ranging from 0.001 to 10 ppm. Each data point is the average value of three replicates. e) Comparison of previously reported chlorpyrifos pesticide detection technologies with our extended‐gate OFET pesticide sensor in terms of the LOD. f) Selectivity of the chlorpyrifos pesticide sensor. g) Response time and recovery time of the pesticide sensor to 1 ppb chlorpyrifos.

The urgent need to increase agricultural productivity while minimizing pesticide contamination necessitates advanced detection methodologies crucial for precision farming and public health protection.^[^ ^34^, ^35^ ^]^ We developed an extended‐gate type OFET based on the stretchable transistors for selective chlorpyrifos detection (**Figure** **6**a). The sensing mechanism relies on the voltage modulation between the extended‐gate electrode and OFET channel, inducing measurable variations in source‐drain currents. To realize specific recognition, a multi‐stage electrode modification process was implemented: the extended‐gate working electrode (GCE) was first coated with chitosan‐functionalized multi‐walled carbon nanotubes (MWCNTs‐CS), subsequently immobilized with acetylcholinesterase (AChE), and finally protected by a Nafion film (Figure 6b). Comprehensive electrochemical characterization through cyclic voltammetry (CV) and electrochemical impedance spectroscopy (EIS) revealed distinct substrate modification effects. As illustrated in Figure 6c, bare GCE exhibited well‐defined REDOX peaks, while MWCNTs‐Cs/GCE showed an enhanced current response due to improved conductivity. AChE/MWCNTs‐Cs/GCE displayed a significant current decrease, confirming successful enzyme immobilization. EIS analysis showed matching charge transfer resistance changes, providing cross‐validation through two complementary electrochemical techniques (Figure S21, Supporting Information).

To ensure sensor reliability, we carried out a cyclic scanning test on the OFET devices (Figure S22, Supporting Information). The devices exhibited excellent repeatability, a critical prerequisite for long‐term sensing applications. Sensing response characteristics to chlorpyrifos revealed a linear dynamic range governed by the equation Δ*I*_DS_/*I*_DS_ = 8.995 lg[c/ppm] + 41.012 (R^2^ = 0.997), yielding a calculated limit of detection (LOD) of 0.032 ppb (calculated as three times the signal‐to‐noise ratio) (Figure 6c,d). Comparative analysis demonstrated that this LOD surpasses most conventional pesticide detection methods (Figure 6e).^[^ ^36^, ^37^, ^38^, ^39^, ^40^ ^]^ Selectivity detection against common agricultural interferents‐glucose, citric acid, urea, Na^+^, Mg^2+^, Al^3+^, SO_4_^2−^, and NO_3_^−^ showed negligible signal deviation (<2%, Figure 6f), confirming excellent selectivity. To further validate the practical applicability, we employed the labeling recovery method to assess its capability to detect pesticide residues in actual samples. The recovery rate of the poisoned water ranged from 98.77% to 124.2%, with minor deviation (Figure S23, Supporting Information), confirming the sensor's reliability for trace pesticide quantification in complex environmental matrices. To evaluate reproducibility and device‐to‐device variability of OFET sensors, pesticide detection was performed on both a single device (five cycles) and 12 OFET sensors. The single device showed excellent reproducibility toward 10 ppb chlorpyrifos, with response values tightly distributed ≈26.5 ± 0.4% (Figure S24, Supporting Information), confirming high sensing reproducibility. Measurements across 12 OFET sensors at 10 ppm chlorpyrifos exhibited a low RSD of 1.62% in sensing response, demonstrating outstanding device‐to‐device uniformity (Figure S25, Supporting Information). Additionally, the pesticide sensor exhibited a fast response time of 6.4 s and a recovery time of 26.6 s toward 1 ppb chlorpyrifos (Figure 6g). To evaluate the OFET sensor's suitability for long‐term pesticide detection, we conducted stability tests on the fabricated sensor. The sensor maintained 98% of its original response to 10 ppm chlorpyrifos even after 16 days of storage (Figure S26, Supporting Information). A comparison of the performances of literature‐reported state‐of‐the‐art pesticide sensors is illustrated in Table S3 (Supporting Information). The fabricated sensor device exhibits superior sensing performance, characterized by the fastest response/recovery time, and good long‐term operational stability against recently reported state‐of‐the‐art pesticide sensors. Additionally, the OFET sensor exhibited conformal adhesion to fruits and vegetables, maintaining performance under deformation, and enabling long‐term monitoring (Figure S27, Supporting Information). With its high sensitivity and accuracy, this stretchable OFET platform offers a versatile tool for precision agriculture.

## 3 Conclusion

In summary, we have demonstrated that the small‐molecule TeNF serves as an effective additive for enhancing the stability of semiconductor polymer without compromising high mobility. The fully stretchable organic transistor, utilizing the TeNF/IDTBT blend film, achieved a maximum mobility of 3.50 cm^2^ Vs^−1^ and an average mobility of 3.30 cm^2^ Vs^−1^. This transistor maintained robust electronic properties even under 100% strain and exhibited consistent performance after 1000 cycles of 30% strain. Furthermore, the TeNF/IDTBT blend OFET demonstrated significant stability improvement, as evidenced by its performance during cyclic scanning, bias stressing, and current switching tests, with reliable storage for up to two months in the air. Based on the high stability and mobility of the OFET, the extended‐gate strategy was adopted to successfully realize the high‐sensitivity detection of chlorpyrifos. The stretchable OFET sensor, with its high stability and performance, holds promise for large‐scale integration and is expected to have widespread applications in diverse sensing platforms.

## 4 Experimental Section

### Materials

The conjugated polymer IDTBT, DPPDTT and PDVT‐10 were purchased from Solarmer Beijing Inc. The conjugated polymer TDPP‐Se was synthesized as described earlier.^[^ ^41^ ^]^ The TeNF was synthesized according to previously reported methods.^[^ ^42^ ^]^ The styrene ethylene butylene styrene elastomers (SEBS H1052 and SEBS H1062) were supplied by Asahi Kasei (Japan). SEBS H1052 with 60 mg mL^−1^ was employed to fabricate the stretchable dielectric layer. SEBS H1062 with 200 mg mL^−1^ was used as the stretchable substrate. The dispersed CNTs aqueous solution was purchased from Chengdu Zhongke Times Nano Energy Tech Co., Ltd for the fabrication of gate, source, and drain electrodes. The OTS solution and anhydrous chlorobenzene were purchased from Sigma Aldrich. MWCNTs powder was purchased from Chengdu Zhongke Times Nano Energy Tech Co., Ltd. High‐density chitosan was supplied by Macklin. AChE was purchased from Sigma Aldrich. PBS buffer fluid was purchased from Solarbio. Standard solution of chlorpyrifos (100 ppm, toluene) was supplied by Tan‐Mo Technology Co., Ltd. All solvents, including isopropanol, acetone, trichloromethane, n‐hexane, O‐1,2‐dichlorobenzene, toluene, and acetic acid, were purchased from commercial sources and used without any purification.

### Thin‐Films Preparation

The Si/SiO_2_ and bare Si substrates were exposed to oxygen plasma (80 W) for 10 min and then modified with OTS at 120 °C for 2 h. Then, the Si/SiO_2_ and bare Si substrates were sonicated successively in chloroform, n‐hexane, and isopropanol at 40 w for 10 min each. IDTBT and DPPDTT were dissolved in anhydrous chlorobenzene (5 mg mL^−1^, 60 °C, 12 h), whereas TDPP‐Se and PDVT‐10 required o‐dichlorobenzene (5 mg mL^−1^, 90 °C, 12 h) for complete dissolution. The TeNF additive solution (2 mg mL^−1^) was dissolved in anhydrous chlorobenzene. The concentration of TeNF in the blend solution was expressed as a molar percentage (mol%) relative to the IDTBT monomer. For example, to prepare 1, 3, 5, and 10 mol% TeNF/IDTBT solutions, 6.9, 20.9, 34.7, and 69.6 µL of a 2 mg mL^−1^ TeNF solution were added to 1 mL of a 5 mg mL^−1^ IDTBT solution, respectively. The blend solution was stirred at 60 °C for 6 h and then coated on OTS‐treated Si/SiO_2_ wafers using the solution shearing method. Subsequently, the prepared organic semiconductor films were annealed on a hot table at 150 °C for 10 min to remove the residual solvent.

### Fully Stretchable Transistor Fabrication and Characterization

OFETs in this work adopt a bottom‐gate top‐contact configuration. The dielectric layers were prepared by spinning coating 60 mg mL^−1^ SEBS H1052 toluene solution on the OTS‐modified Si/SiO_2_ wafers at a speed of 1000 rpm for 50 s, followed by annealing at 120 °C for 10 min. For the source/drain electrodes, the CNTs solution was directly spray‐coated onto the Si wafer. The gate electrodes were prepared by spray‐coating the CNTs solution on the Si wafer, and then 200 mg mL^−1^ of SEBS H1062 toluene solution was poured on it as the stretchable substrate. Finally, the intrinsically stretchable organic field‐effect transistor with BG‐TC configuration was fabricated by sequentially transferring SEBS dielectric, TeNF/IDTBT semiconductor film, and CNTs source/drain electrodes onto the SEBS stretchable substrate with the gate electrode from their Si/SiO_2_ wafer.

### Extended‐Gate OFET Pesticide Sensor

The extended‐gate electrode used a commercial screen‐printed electrode. 1 mL 10 mg mL^−1^ chitosan solution was prepared with 2% acetic acid solution, and after it was completely dissolved, the MWCNTs‐Cs mixed solution was obtained by adding 2 mg MWCNTs. First, 5 µL of the prepared MWCNTs‐Cs solution was coated on the working electrode of the extended‐gate electrode and air‐dried naturally to obtain an MWCNTs‐Cs/GCE electrode. Then, 6 µL 0.02 u µL^−1^ AchE (1 mg mL^−1^ PBS) was coated on the MWCNTs‐Cs/GCE electrode surface and dried at room temperature to obtain an AChE/MWCNTs‐Cs/GCE electrode. The modified electrodes were stored at 4 °C for further use. Finally, the obtained stretchable field‐effect transistor device was connected to the modified extended‐gate electrode using gallium‐silver alloy and gold wire for the test of the organophosphorus pesticide chlorpyrifos.

### Characterization

The electrical characteristics of the stretchable organic transistors were measured using a Keithley 4200 semiconductor characterization system under ambient conditions. The capacitances of the dielectric were measured using Analyzer mode (Chemical impedance analyzer IM 3590). The LFN measurements were performed using a PDA FS380 semiconductor parameter analyzer. The electrochemical workstation was used to verify the successful modification of the extended‐gate electrode. The OSC film morphology was examined using AFM (Bruker Dimension ICON‐PT) with a scanning scope of 500 × 500 nm. XPS was performed using a Thermo Scientific K‐Alpha instrument from America. UV–Vis–NIR absorption spectroscopy was performed on a PE Lambda 750 with a polarizer (Japan). EPR experiments were conducted using a Bruker EMXplus EPR spectrometer at 100 K. Raman spectra were performed using Horiba LabRAM HR Evolution (Japan), with a wavelength of the excitation laser source of 532 nm. GISAXS data were collected through the EIGER 2R1M detector, characterized by a pixel size of 75 µm × 75 µm and a total resolution of 1028 × 1062 pixels, and the X‐ray wavelength (λ) used was 1.54 Å.

### DFT Calculation

First, IDTBT and TeNF were subjected to energy minimization in Chem3D, respectively. It was then optimized by the wb97xd/6‐31g(d,p) level of the Gaussian 16 program to obtain lower energy stable molecules. The optimized molecule is used as the initial conformation before binding, and a possible interaction model is built in GaussView. The possible binding modes within the allowable range of distance and included angle were then obtained. These structures were originally optimized at the wb97xd/6‐31g(d,p) level of the Gaussian16 program to obtain low‐energy conformations.

## Conflict of Interest

The authors declare no conflict of interest.

## Author Contributions

H.Y., X.Y., and W.H. conceived and designed the experiments. W.W. contributed to the investigation, data curation, and transistor preparation. L.D. carried out the theoretical studies. W.W., X.L., and X.Z. contributed to the mechanical and electrical performance of transistors. W.W., H.Y., and X.Z. contributed to the stability and mechanism exploration. W.W. and Y.W. contributed to methodology, conceptualization, and data curation. W.W. wrote the manuscript, and all authors reviewed it.

## Supporting information



Supporting Information

## Data Availability

The data that support the findings of this study are available from the corresponding author upon reasonable request.
