# Supplementary material for: Stabilizing Stretchable Organic Transistors Through Small‐Molecule Additive Blending for Ultra‐Sensitive Pesticide Detection
Source: Adv Sci (Weinh). 2025 Oct 13;12(47):e13397. doi: 10.1002/advs.202513397 (PMC12713046; doi:10.1002/advs.202513397)
Supplement: Supplementary file 1 — Supporting Information [file ADVS-12-e13397-s001.docx]

Supporting Information

Stabilizing Stretchable Organic Transistors through Small-molecule Additive Blending for Ultra-sensitive Pesticide Detection

**Authors**

Weiyu Wang, Liya Dai, Xiangxiang Li, Xiaoying Zhang, Huiqi Yang, Xinran Zheng, Yan Wang, Xin Ye*, Hui Yang*, Wenping Hu

State Key Laboratory of Advanced Materials for Intelligent Sensing, Key Laboratory of Organic Integrated Circuit, Ministry of Education & Tianjin Key Laboratory of Molecular Optoelectronic Sciences, Department of Chemistry, School of Science & Institute of Molecular Aggregation Science, Tianjin University, Tianjin 300072, China

E-mail: yanghui2018@tju.edu.cn

**Supplementary Table 1.** Comparison of previously reported stretchable transistors with our high-performance transistor in terms of mobility and stretchability.


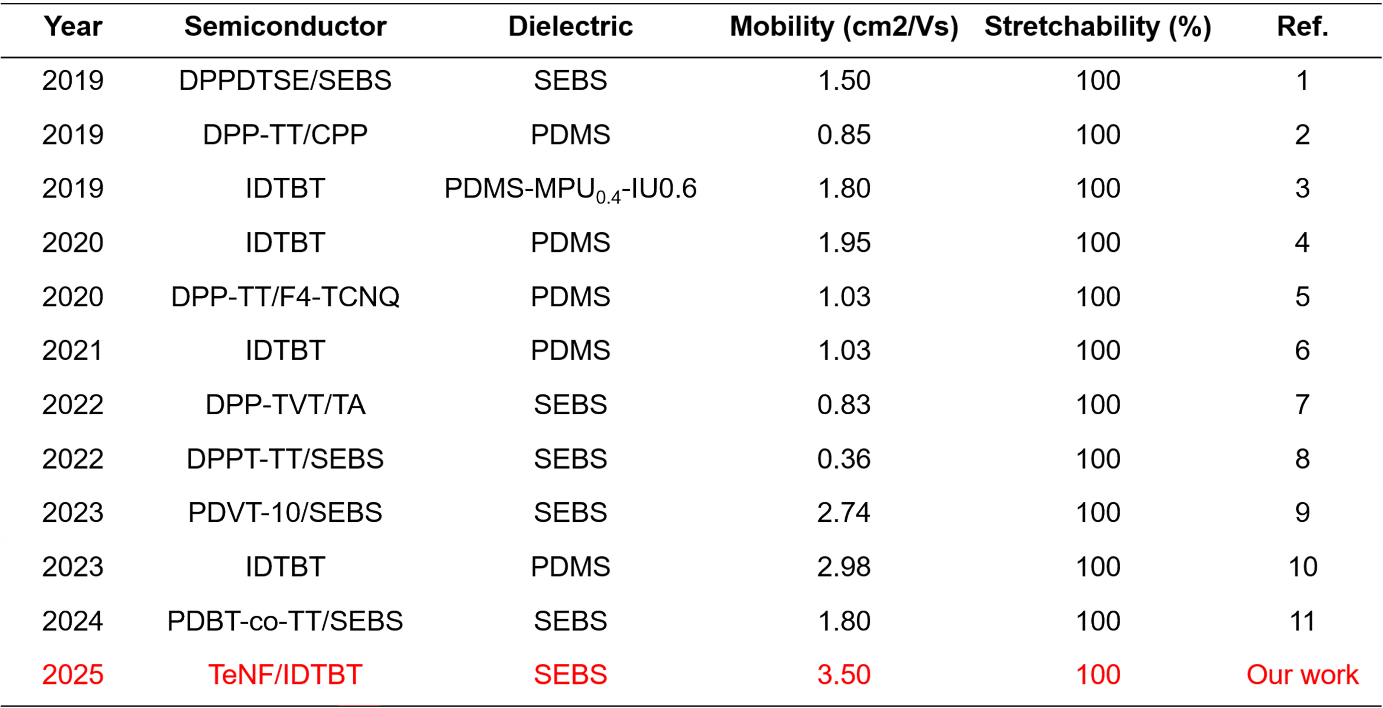


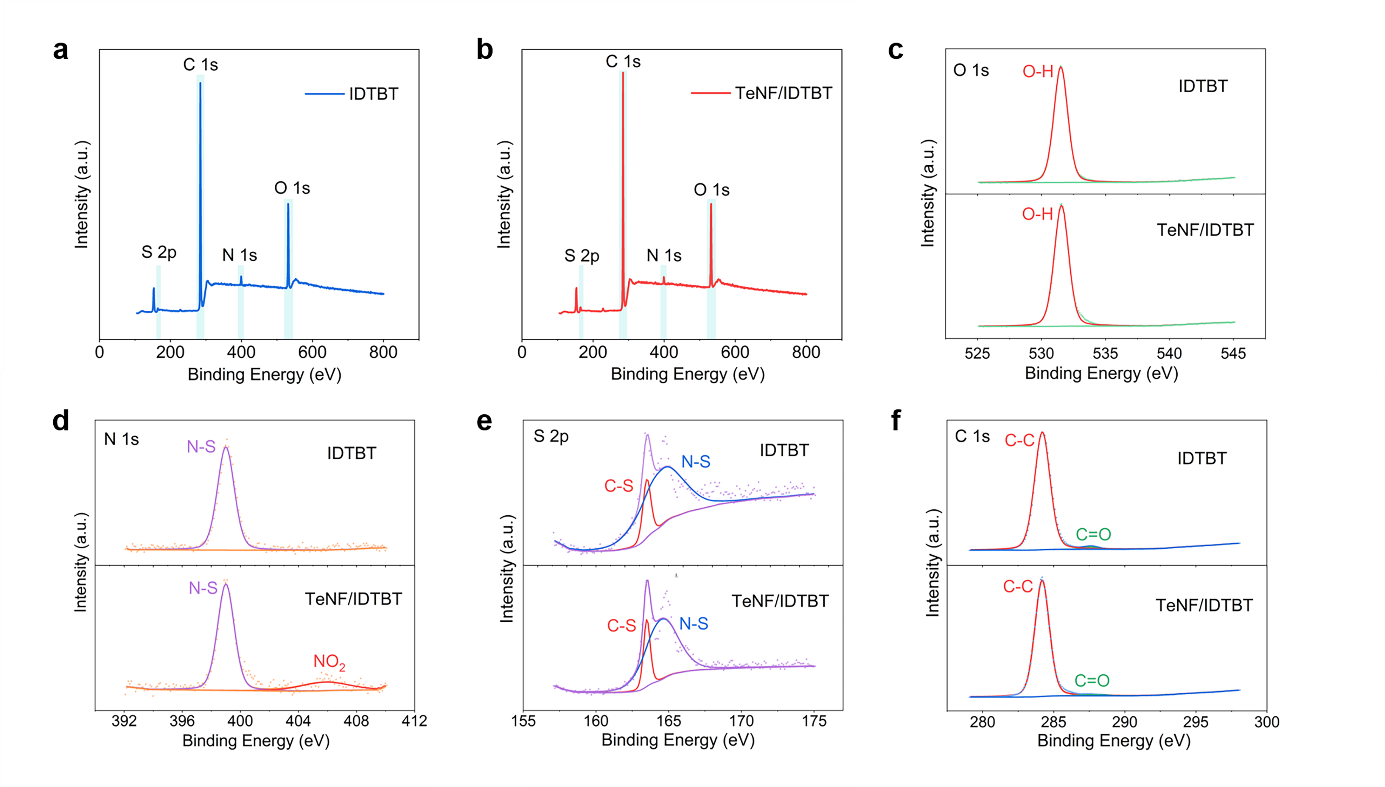


**Figure S1.** a,b) XPS full survey spectra of IDTBT and TeNF/IDTBT blend films. c-f) O 1s, N 1s, S 2p, and C 1s regions of IDTBT and TeNF/IDTBT blend films.


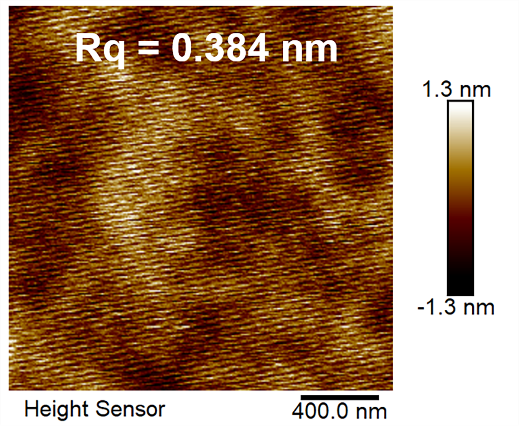


**Figure S2.** AFM image of the SEBS dielectric layer.


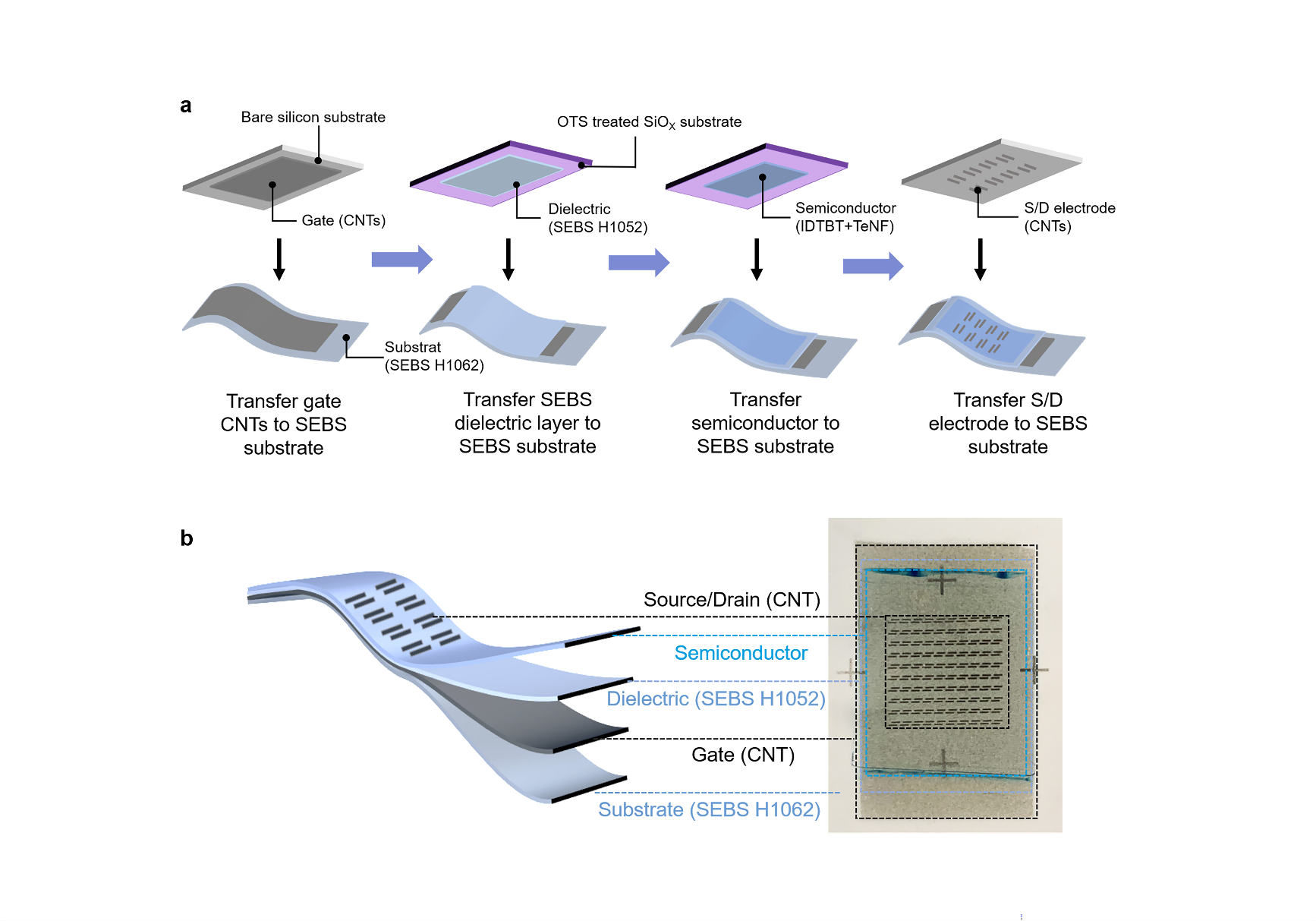


**Figure S3.** a) Diagram illustration of the fabrication process of intrinsically stretchable organic field-effect transistors. Gate, source/drain electrodes were prepared by the mask spraying method on a cleaned Si wafer. Semiconductors were fabricated on OTS-treated Si/SiO_2_ wafer using solution shearing methods. The dielectric layer was constructed onto an OTS-treated Si/SiO_2_ wafer by spin-coating. Finally, the layer-by-layer transfer method was adapted to fabricate stretchable transistors. b) Physical image of the intrinsically stretchable organic transistor. The thickness of the SEBS substrate can be altered from ~10 µm to 1 µm; gate dielectric thickness, 500 nm; capacitance, 1.48 nF cm^−2^; semiconductor thickness, around 15 nm; channel length, 100 μm; channel width, 400 μm.


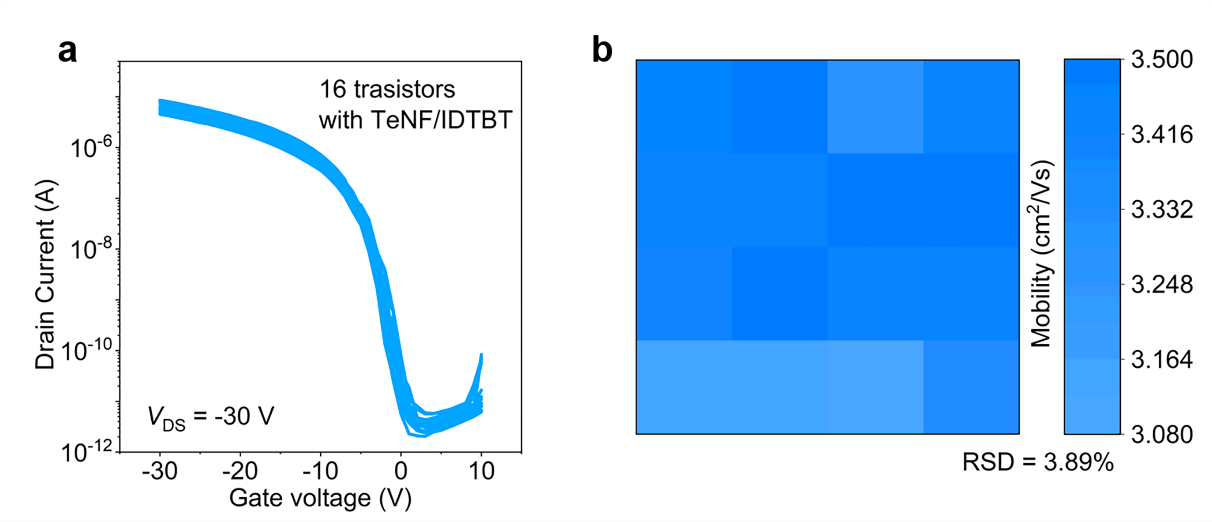


**Figure S4.** Device-to-device uniformity of the 16 OFET devices. a) Transfer curves of the 16 transistors. b,c) Statistical histogram of mobility.


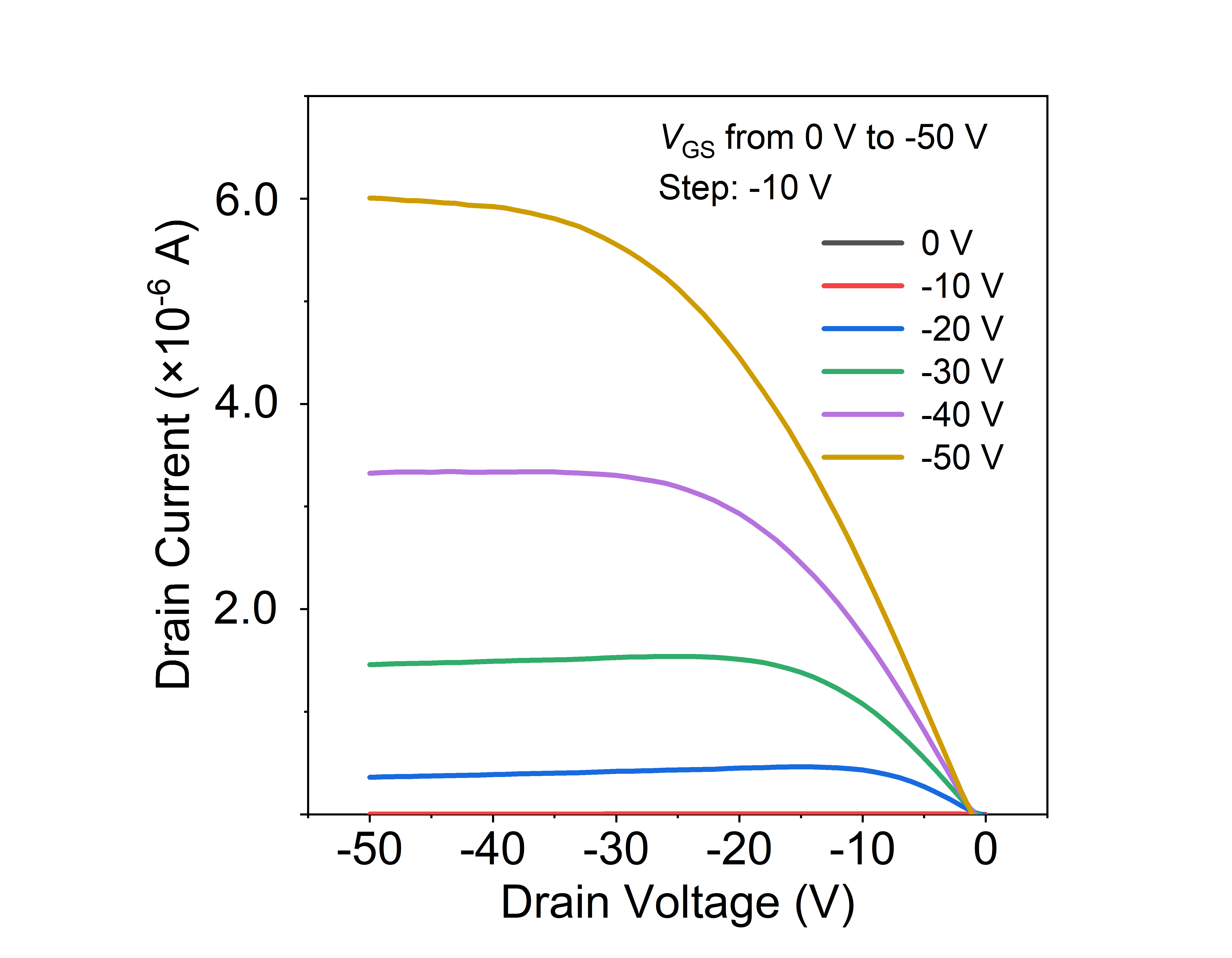


**Figure S5.** A typical output characteristic from an OFET device fabricated using a 3 mol% TeNF/IDTBT blend semiconductor film.


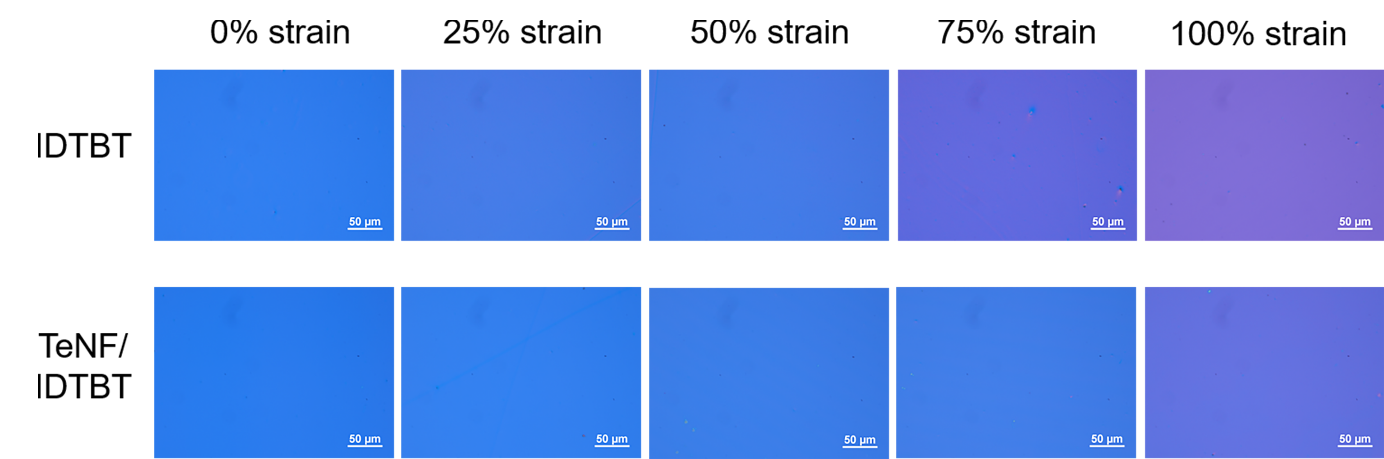


**Figure S6.** Optical microscope images of neat IDTBT film (above) and TeNF/IDTBT blend film (nether) under 0%, 25%, 50%, 75%, 100% strain. Crack on-set strain supported by a PDMS substrate.


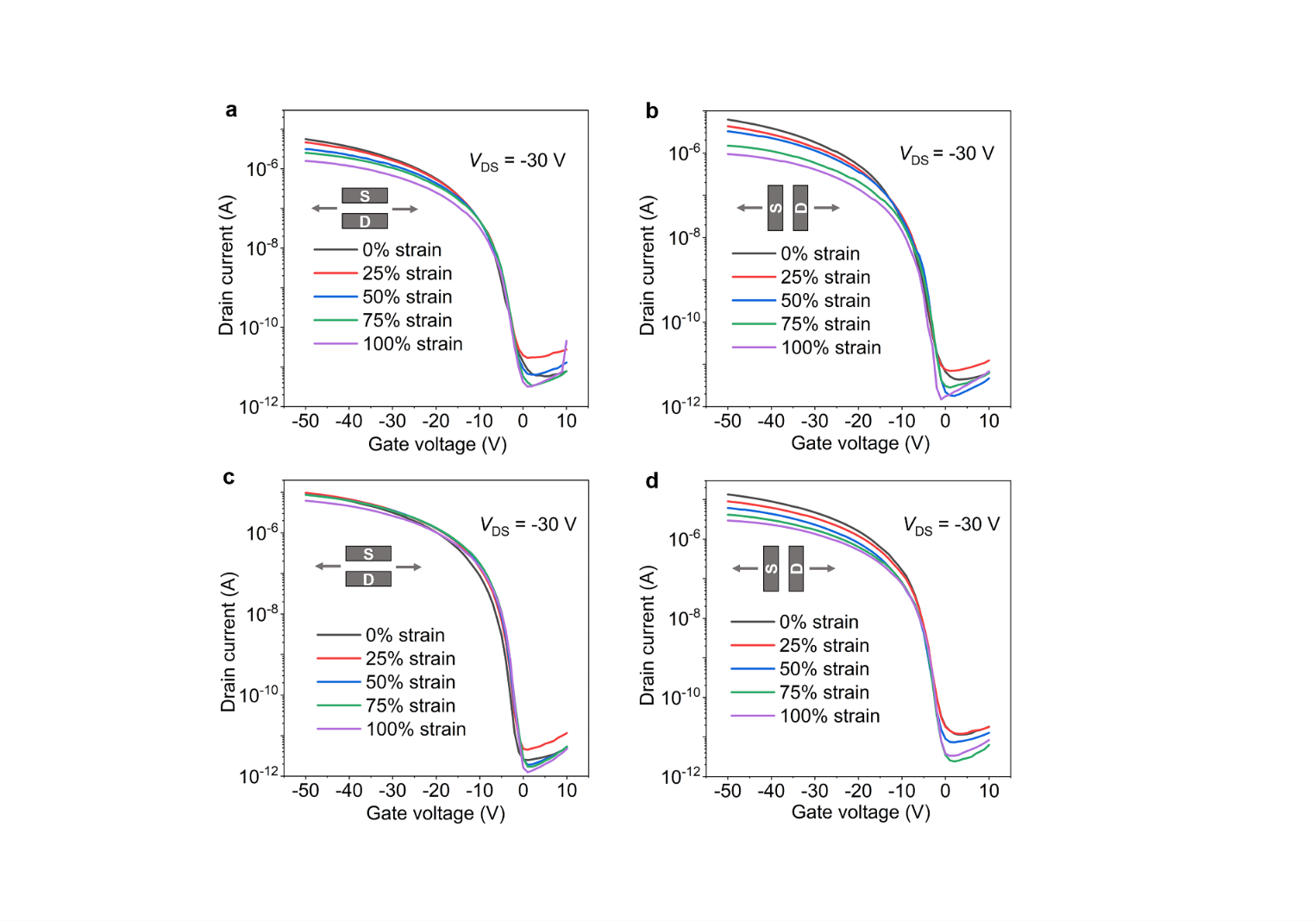


**Figure S7.** a,b) Transfer curves of pristine IDTBT OFETs under various strains, with the charge transport parallel to (a) and perpendicular to (b) stretching direction. c,d) Transfer curves of blend TeNF/IDTBT OFETs under various strains, with the charge transport parallel to (c) and perpendicular to (d) stretching direction.


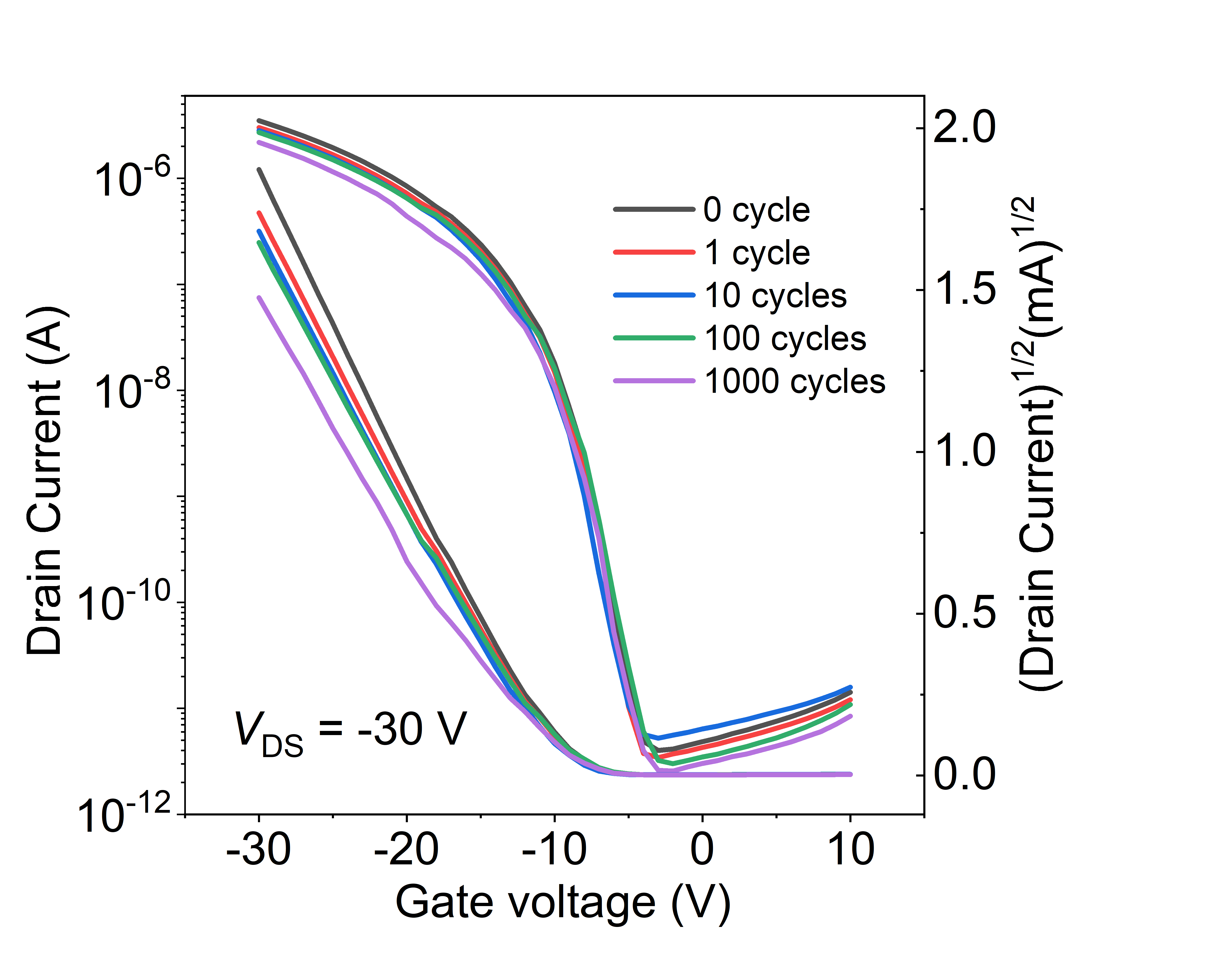


**Figure S8.** Transfer curves of stretchable OFET with varying stretching cycles under 30% strain.


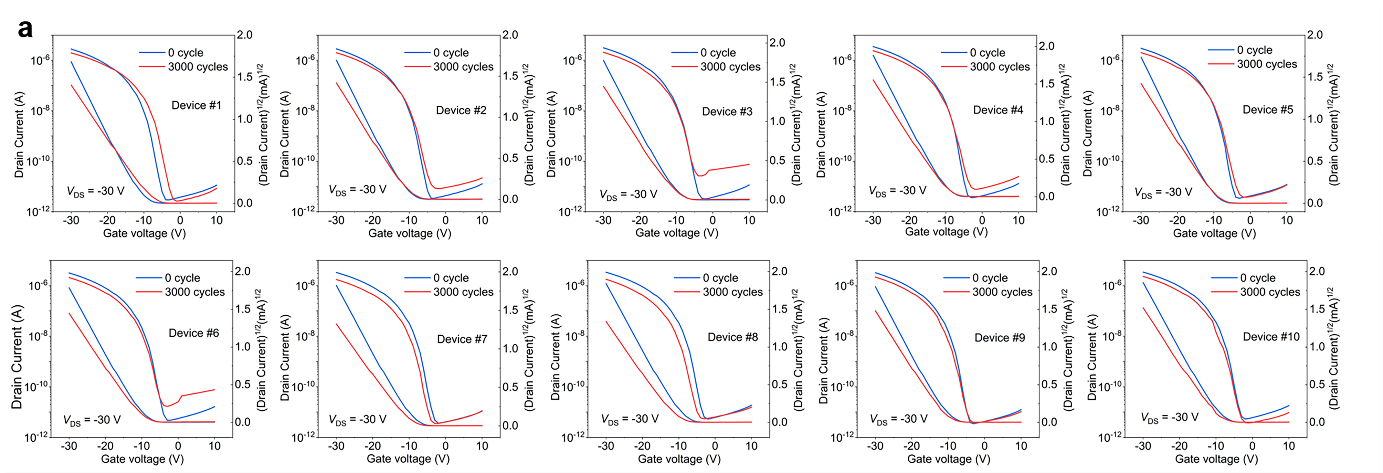


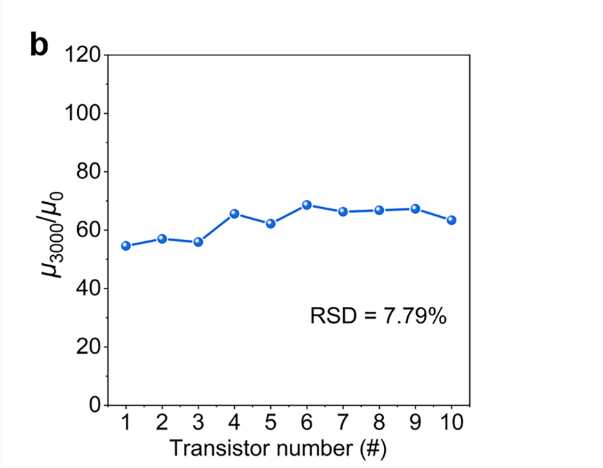


**Figure S9.** Device-to-device uniformity of the 10 OFET devices. a) Transfer curves and *μ*_3000_/*μ*_0_ (b) of 10 devices before and after 1,000 cyclic stretching cycles at 30% strain.

**Supplementary Table 2.** Measured device geometry and dielectric capacitance in the fully stretchable transistor under different strains.


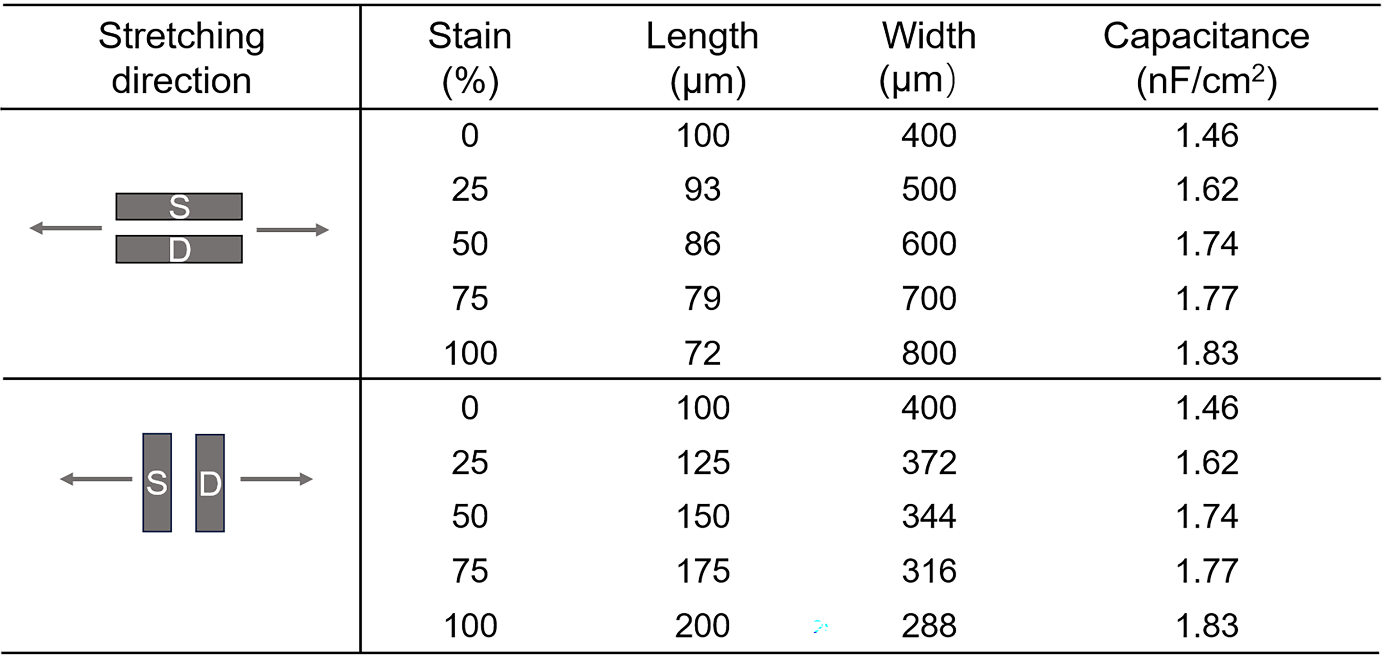


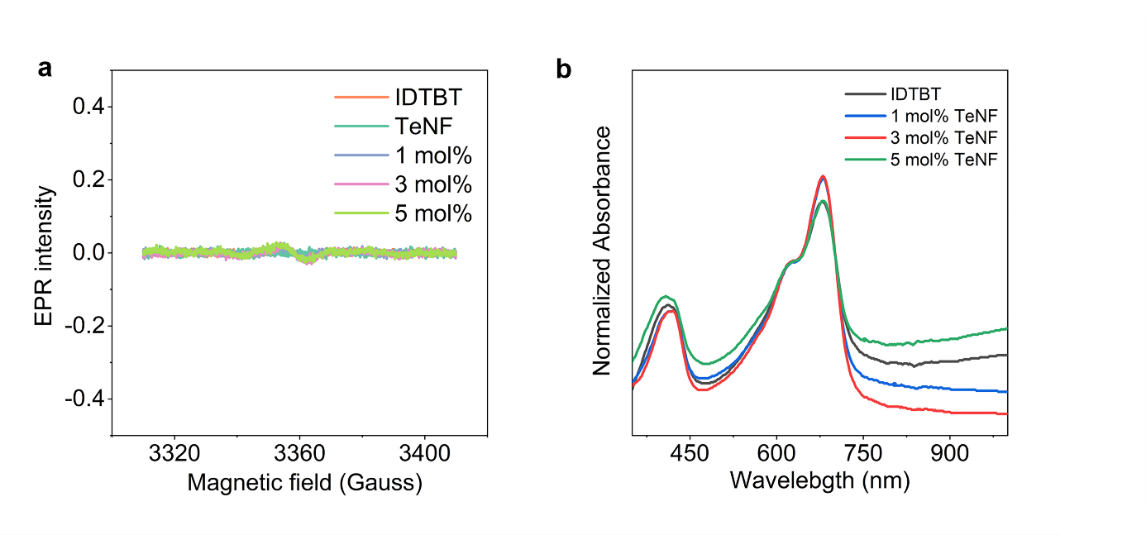


**Figure S10.** a,b) EPR spectra (100 k) (a) and UV-vis-NIR spectra (b) of neat IDTBT films and TeNF/IDTBT blend films.


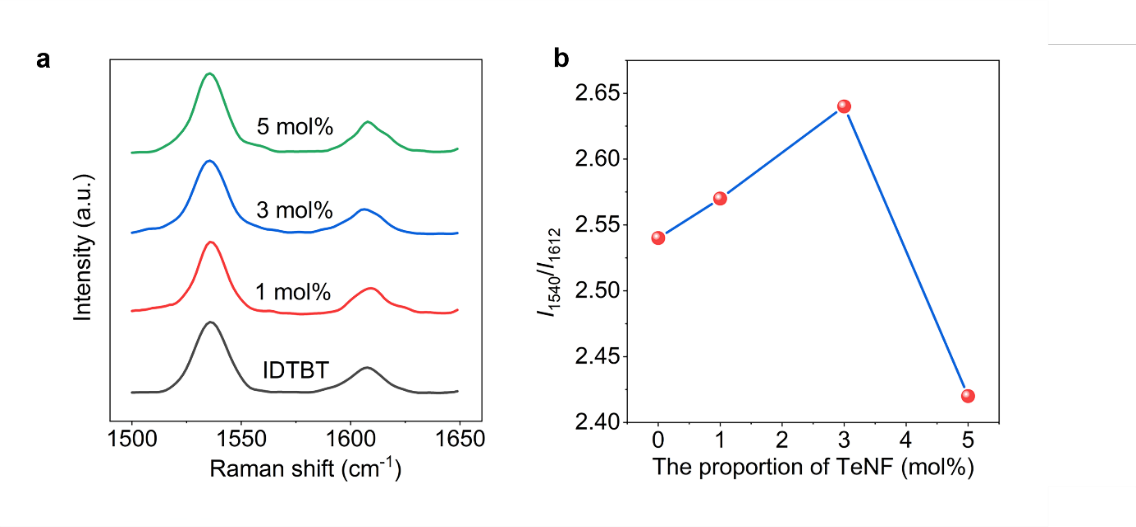


**Figure S11.** a,b) Raman spectra (a) and *I*_1536_/*I*_1608_ values (b) of different semiconductor films. A larger ratio of *I*_1536_/*I*_1608_ indicates better backbone coplanarity.


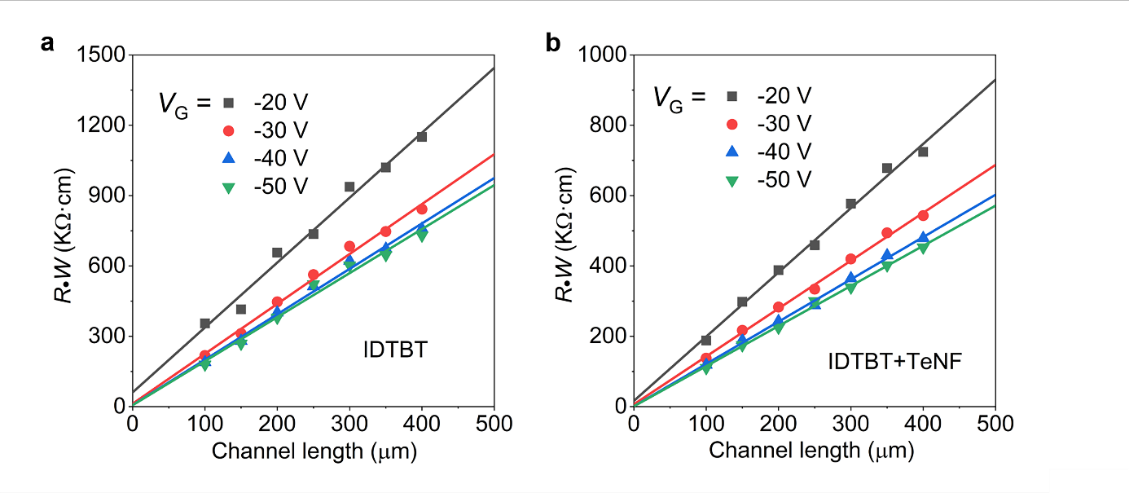


**Figure S12.** a,b) TLM method for the extraction of contact resistance of pristine IDTBT OFET (a) and TeNF/IDTBT blend OFET (b).


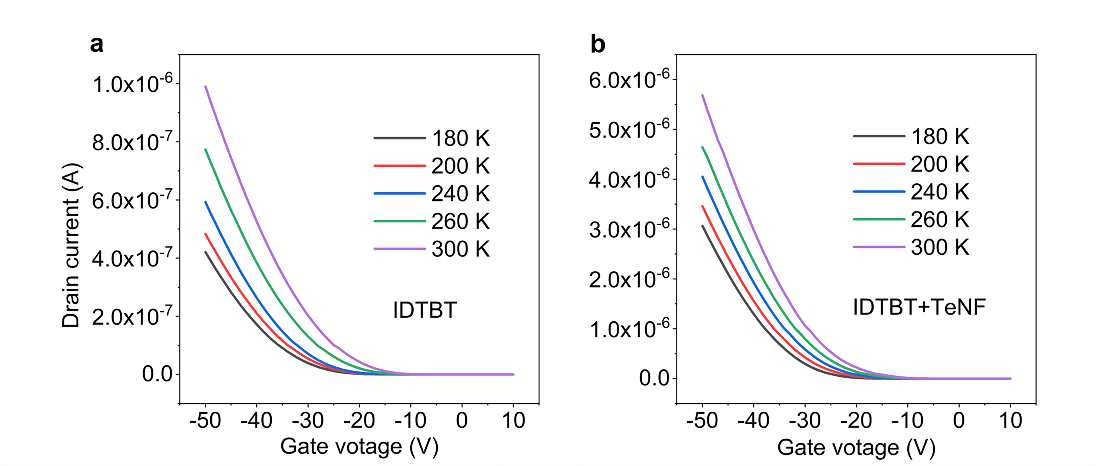


**Figure S13.** a,b) Temperature-dependent transfer characteristics of different semiconducting films: a neat IDTBT film (a), a TeNF/IDTBT blend film (b).

The *E*_A_ in both neat IDTBT films and TeNF/IDTBT blend films was estimated using the Arrhenius equation:

ln(*μ*) = −*E*_A_/k_B_*T*

where k_B_ was the Boltzmann constant, *μ* was the mobility, and *T* was the Kelvin temperature.


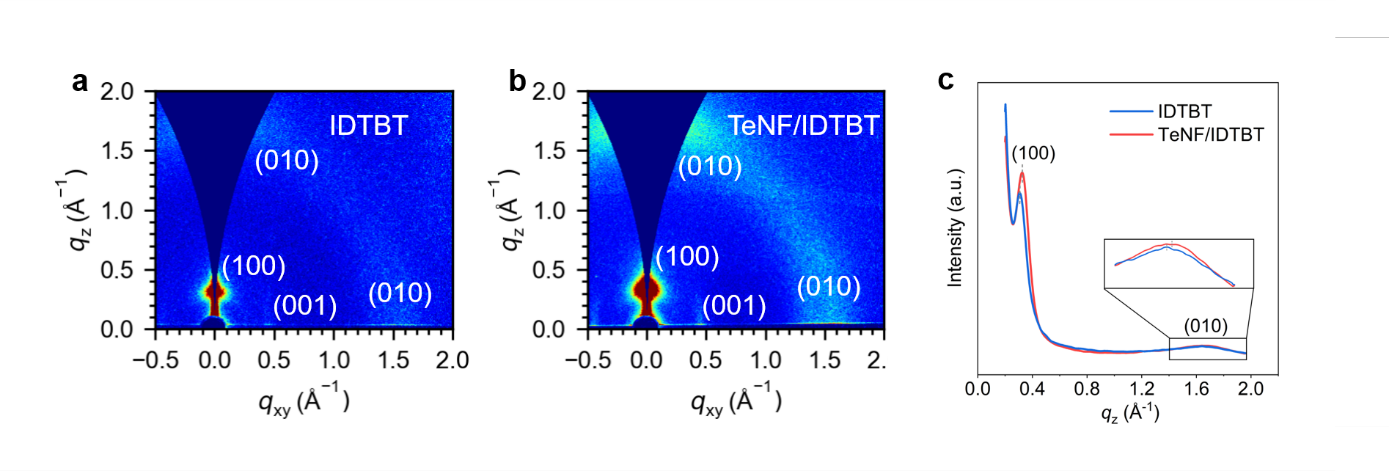


**Figure S14.** a, b) GISAXS images of IDTBT (a) and TeNF/IDTBT (b) films. c) Out-of-plane 1D XRD.


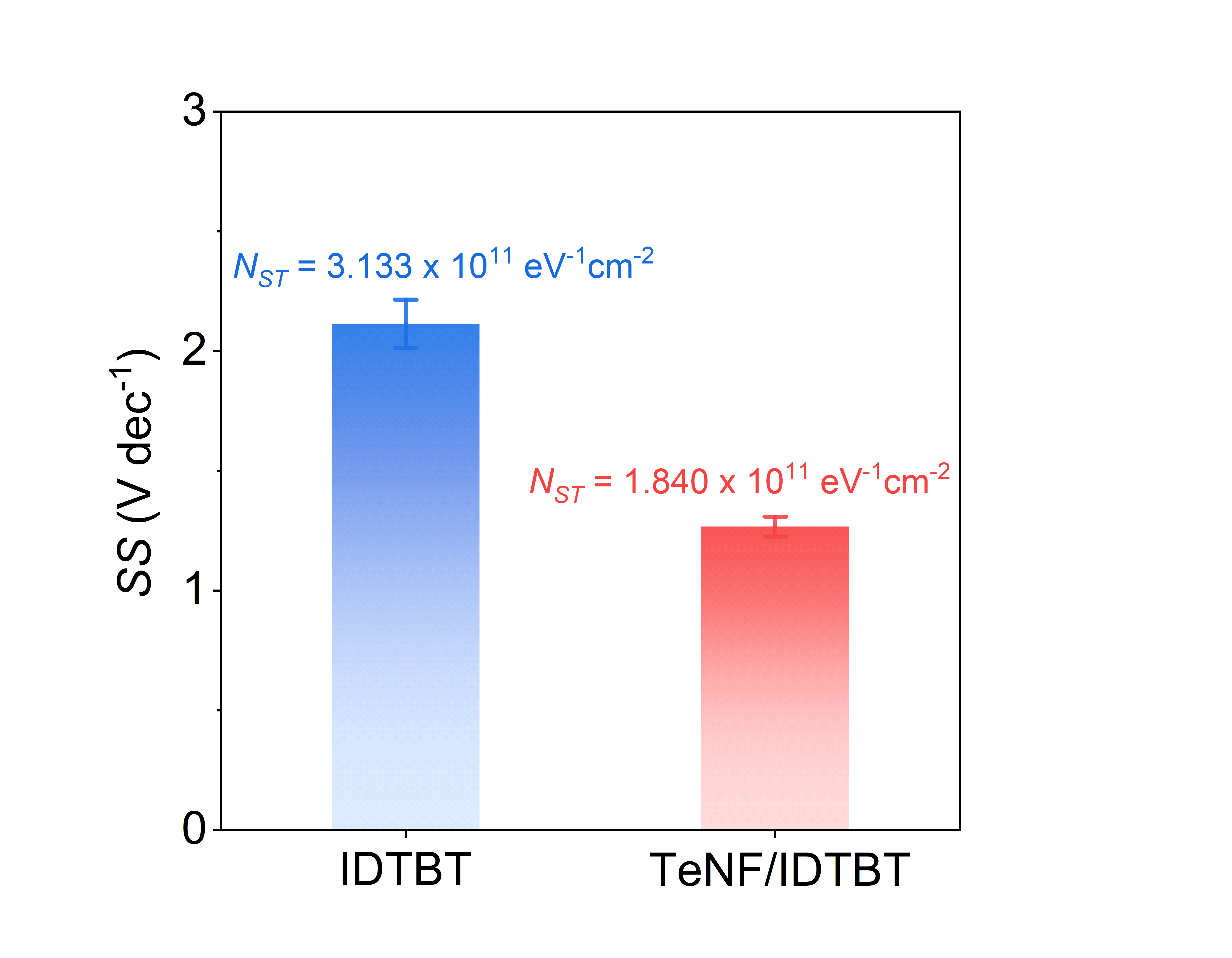


**Figure S15.** The interfacial trap states for IDTBT and TeNF/IDTBT film devices.

The subthreshold swing (*SS*), a critical parameter in transistor performance, is strongly correlated with the interfacial trap state between the semiconductor and dielectric layers. To quantitatively evaluate the interface trap density, we applied the following formula:

$$\text{N}_{\text{S}\text{T}}\text{=}\frac{\text{C}_{\text{dielectric}}}{\text{q}^{\text{2}}}\text{(}\frac{\text{q}\text{SS}}{\text{k}_{\text{B}}\text{T}\text{ln10}}\text{-1)}$$

where *N_ST_* denotes the interface trap density, *k_B_* is the Boltzmann constant, *T* is the temperature in Kelvin, *q* is the elementary charge, and *C*_dielectric_ is the capacitance per unit area of the dielectric.


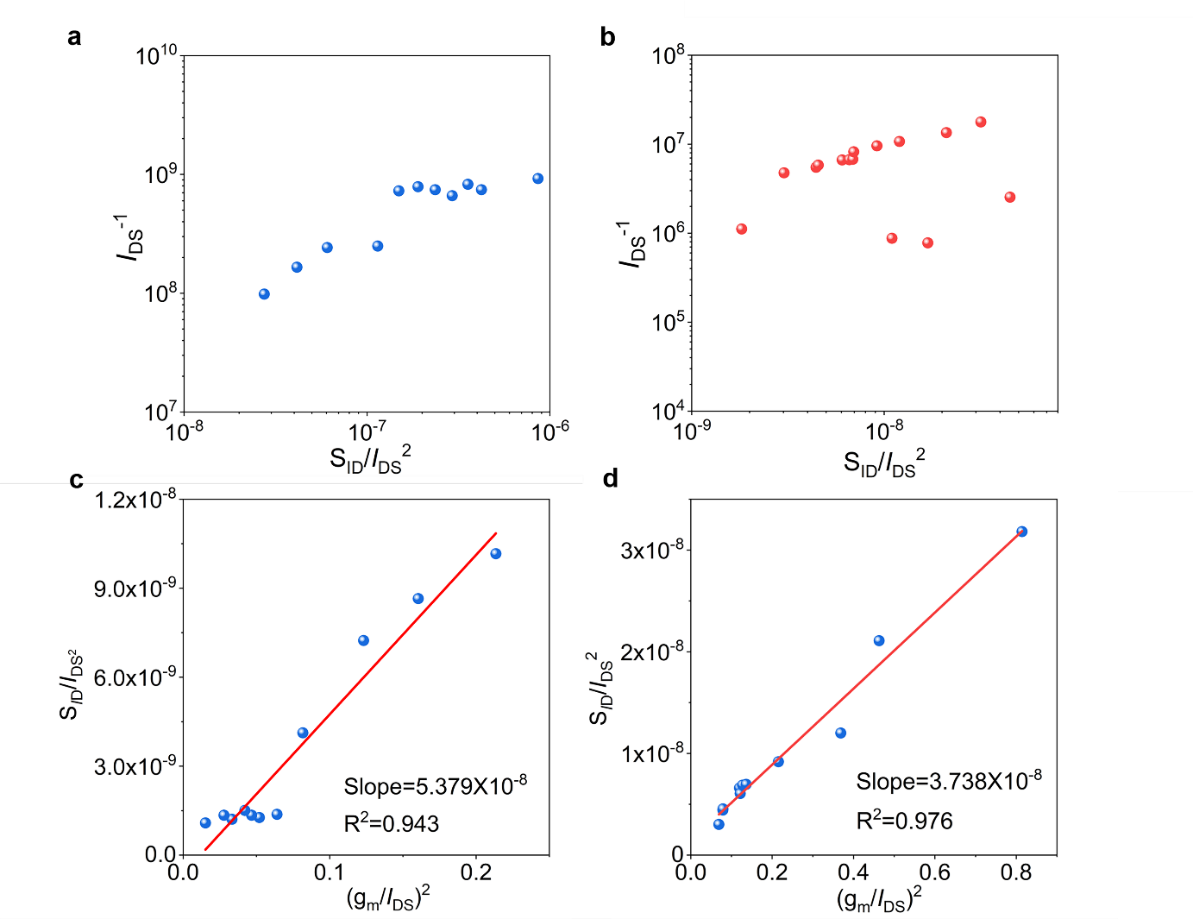


**Figure S16.** a,b) The relationship between *I*_DS_^-1^ of pristine IDTBT OFET (a) and TeNF/IDTBT blend OFET (b). c,d) Linear fitting of *S*_ID_/*I*_DS_^2^ and (*g*_m_/*I*_DS_)^2^ based on pristine IDTBT OFET (c) and TeNF/IDTBT blend OFET (d).

Figure S16a,b showed that *S*_ID_*/I*_DS_^2^ was not proportional to *I*_DS_^-1^, which indicated that the noise model did not conform to the model Δ*μ*. In contrast, the Δn model mainly described the trapping and releasing behavior of carriers at the interface between the semiconductor and the dielectric layer, which could be expressed as

$\frac{\text{S}_{\text{I}\text{D}}}{{\text{I}_{\text{DS}}}^{\text{2}}}\text{=}\frac{\text{q}^{\text{2}}\text{k}\text{T}\text{N}_{\text{ST}}}{\text{WL}{\text{C}_{\text{i}}}^{\text{2}}\text{f}}\text{×}$ ${\text{(}\frac{\text{g}_{\text{m}}}{\text{I}_{\text{DS}}}\text{)}}^{\text{2}}$

where *q* was the electron charge, *k* was the Boltzmann constant, *T* was the Kelvin temperature, *N*_ST_ was the surface trap density, *W* was the product of the channel width, *L* was the product of the channel length, *C*_i_ was the capacitance per unit area of the gate dielectric.


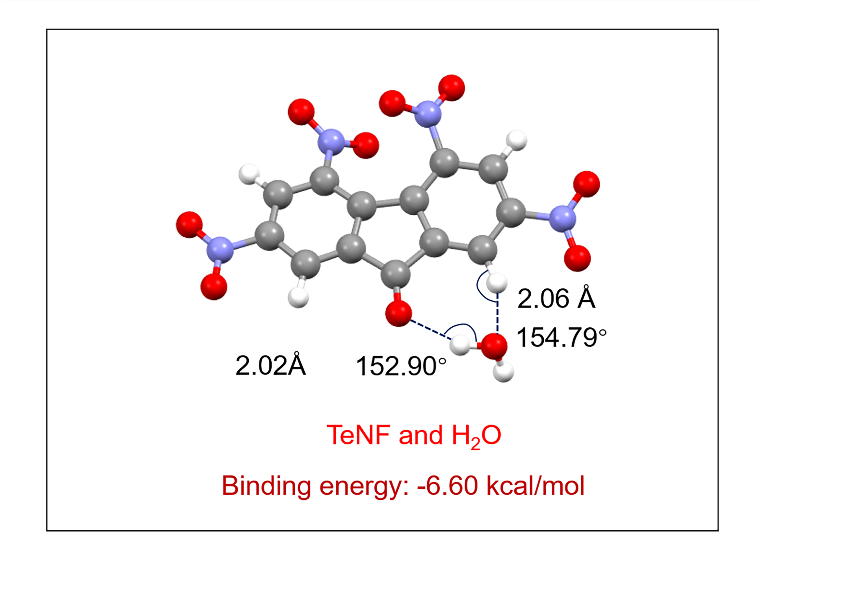


**Figure S17.** Computational evaluation of the interaction between additive and water.


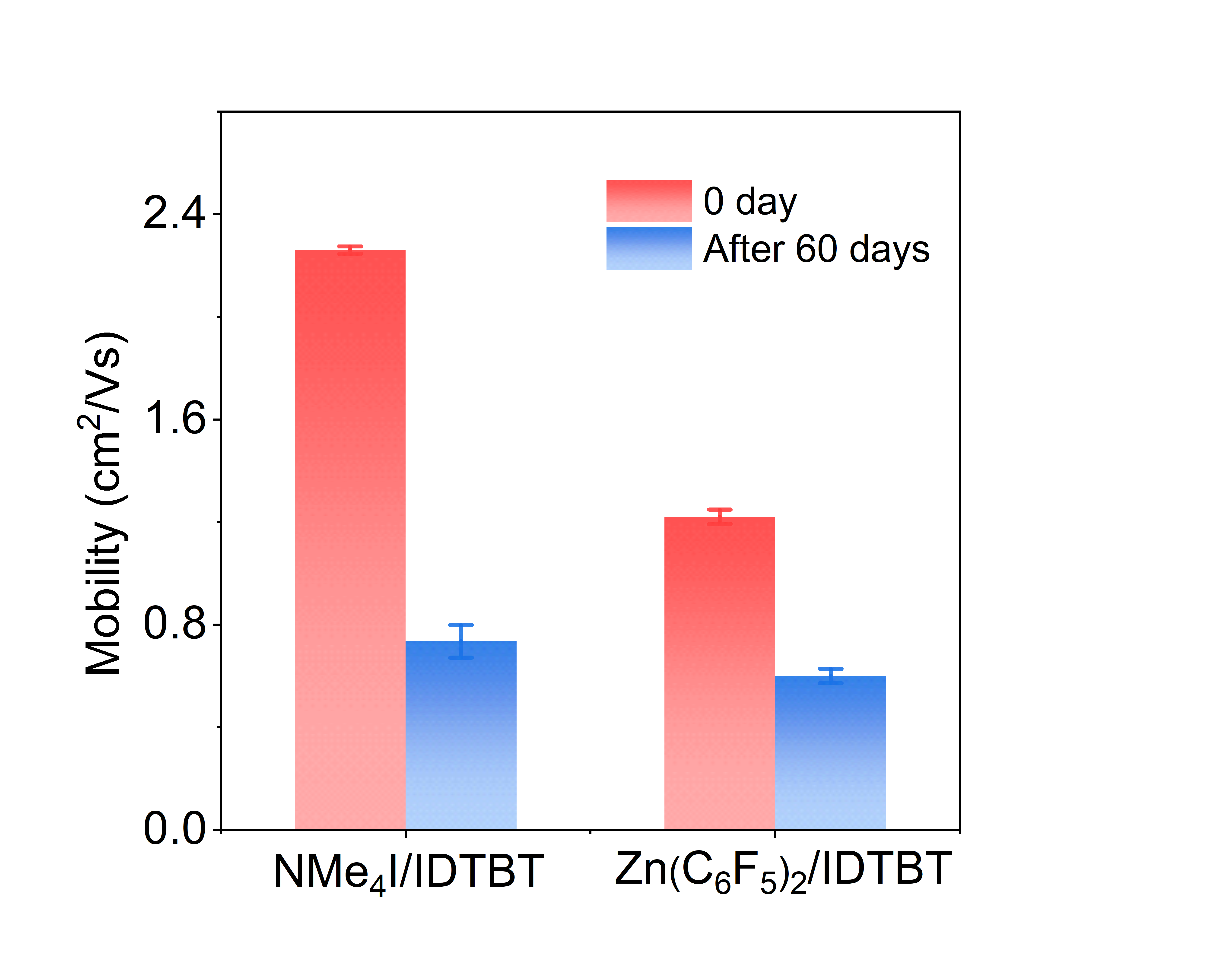


**Figure S18**. Influence of NMe_4_I and Zn(C_6_F_5_)_2_ on environmental stability performance of OFETs devices.

**
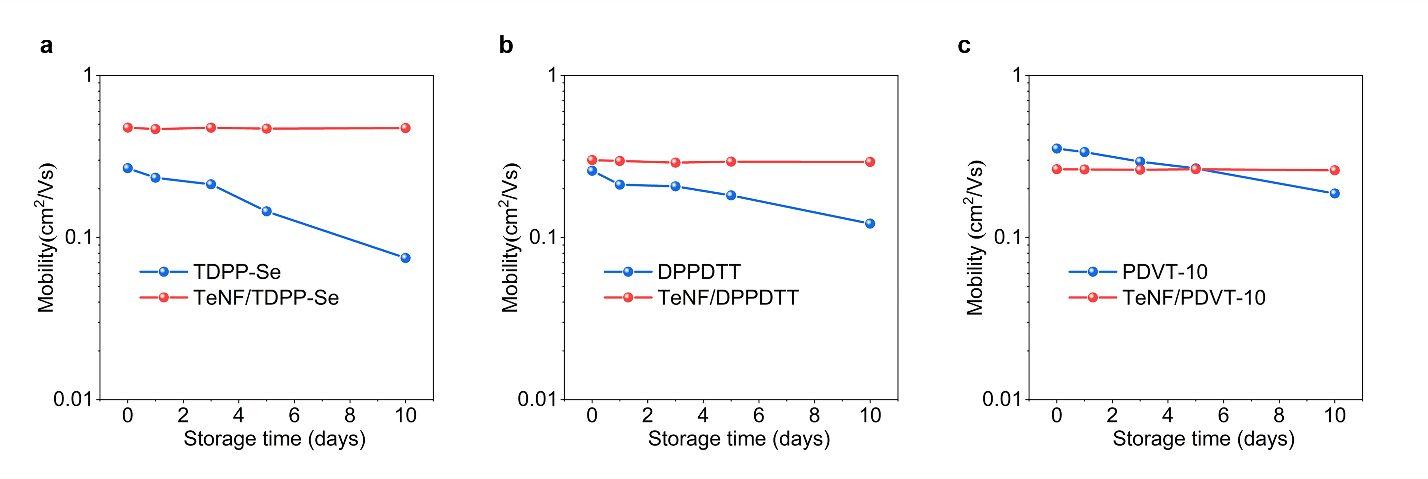
**

**Figure S19.** a-c) Extracted the mobilities of TDPP-Se-based devices (a), DPPDTT-based devices (b), and PDVT-10-based OFET devices (c) over varying storage times.


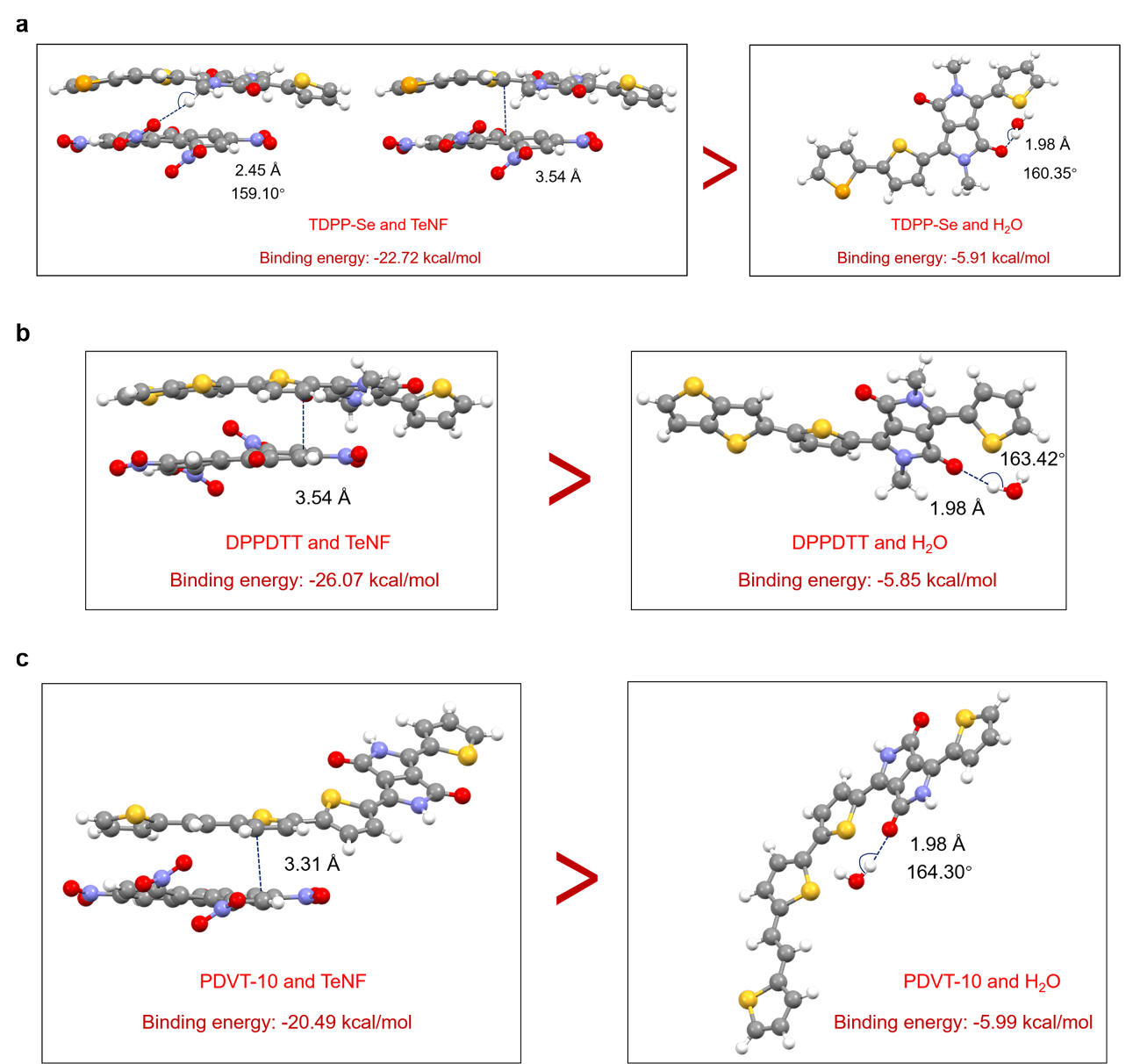


**Figure S20.** a-c) Computational evaluation of the interactions between polymer backbone (TDPP-Se (a), DPPDTT (b), PDVT-10 (c)) and additive, polymer backbone (TDPP-Se (a), DPPDTT (b), PDVT-10 (c)) and water based on DFT.


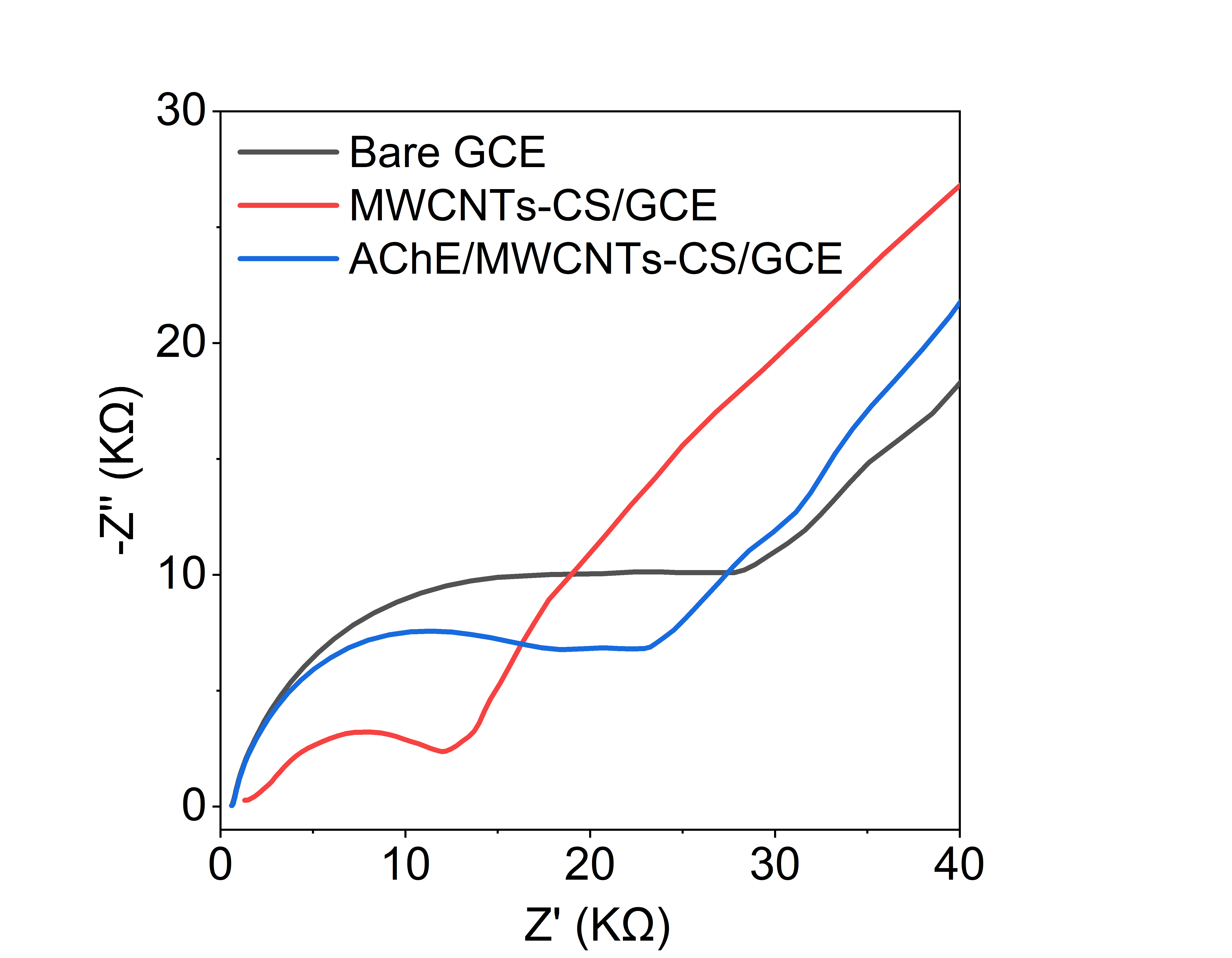


**Figure S21.** Electrochemical impedance spectroscopy (EIS) curve of bare GCE and modified GCE.

The electrochemical behaviors of bare GCE and modified GCE were studied with EIS. As shown in Figure S21, the semi-circular diameter in the Nyquist plot represented the electron transfer resistance of the electrode. The larger the semi-circle diameter of the EIS curve, the larger the resistance value of the corresponding electrode. And the results mentioned above also demonstrated the successful immobilization of each additional layer.


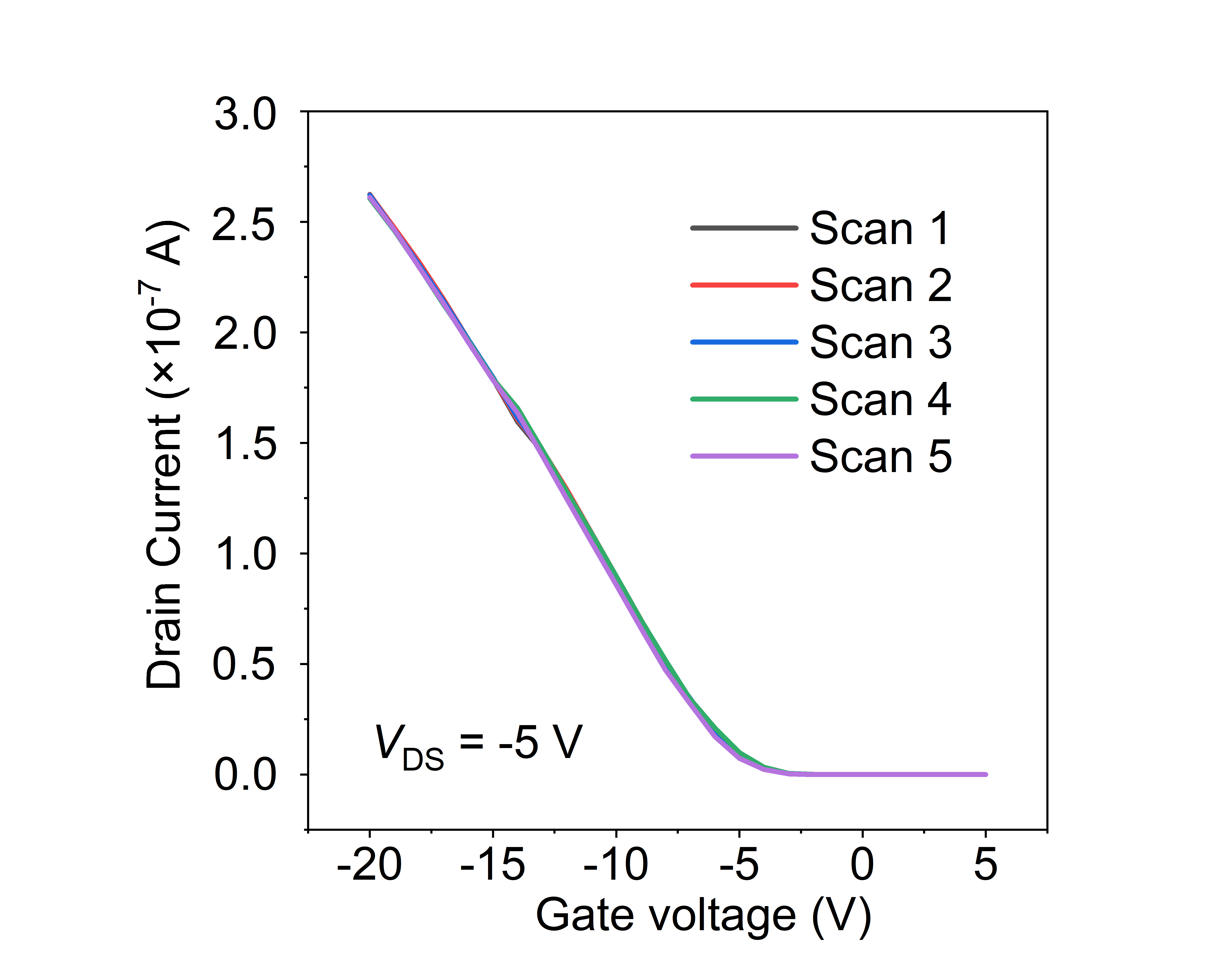


**Figure S22.** Cyclic scanning test conducted before pesticide sensing.


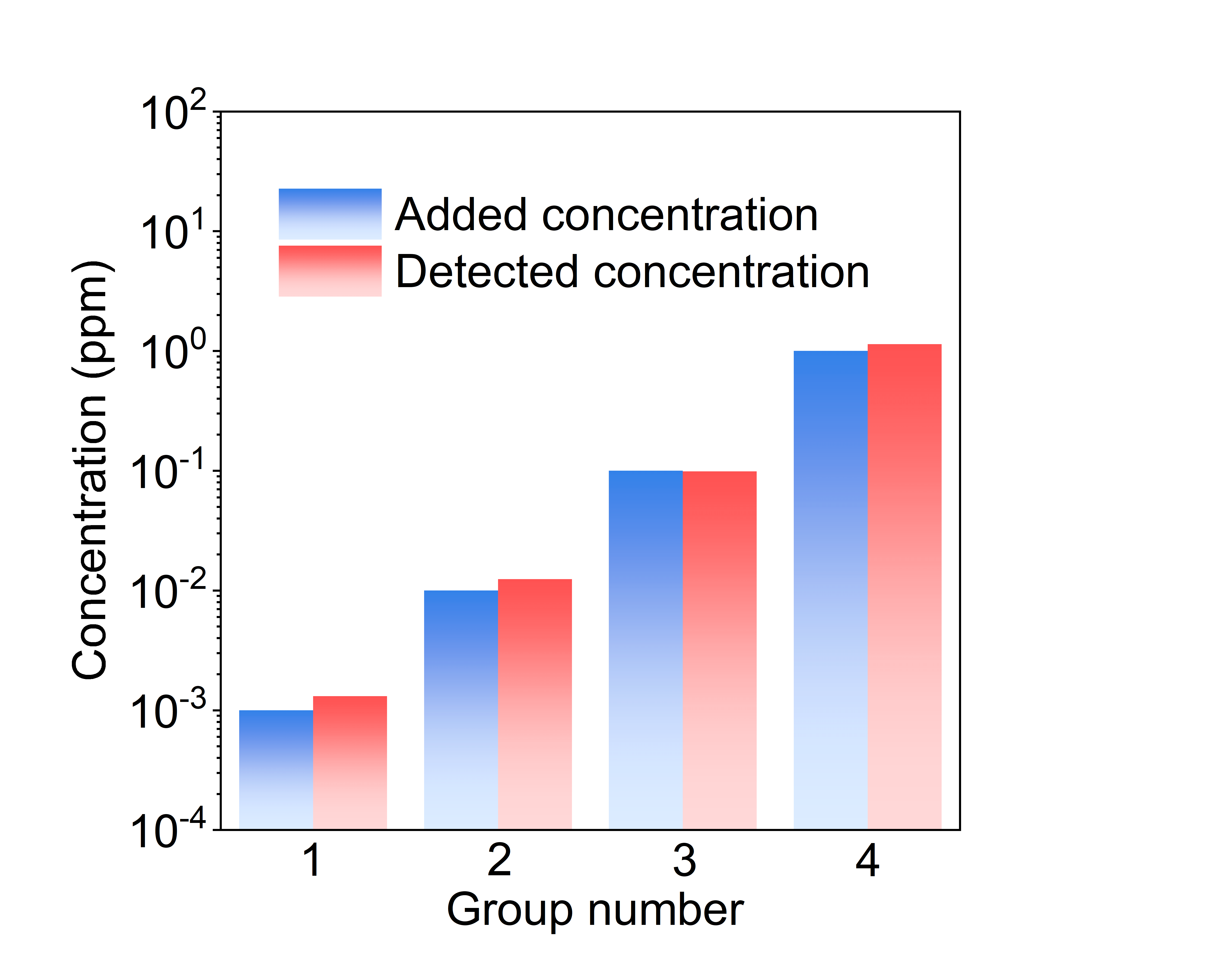


**Figure S23.** The labeling recovery method for pesticide sensors based on stretchable transistor.


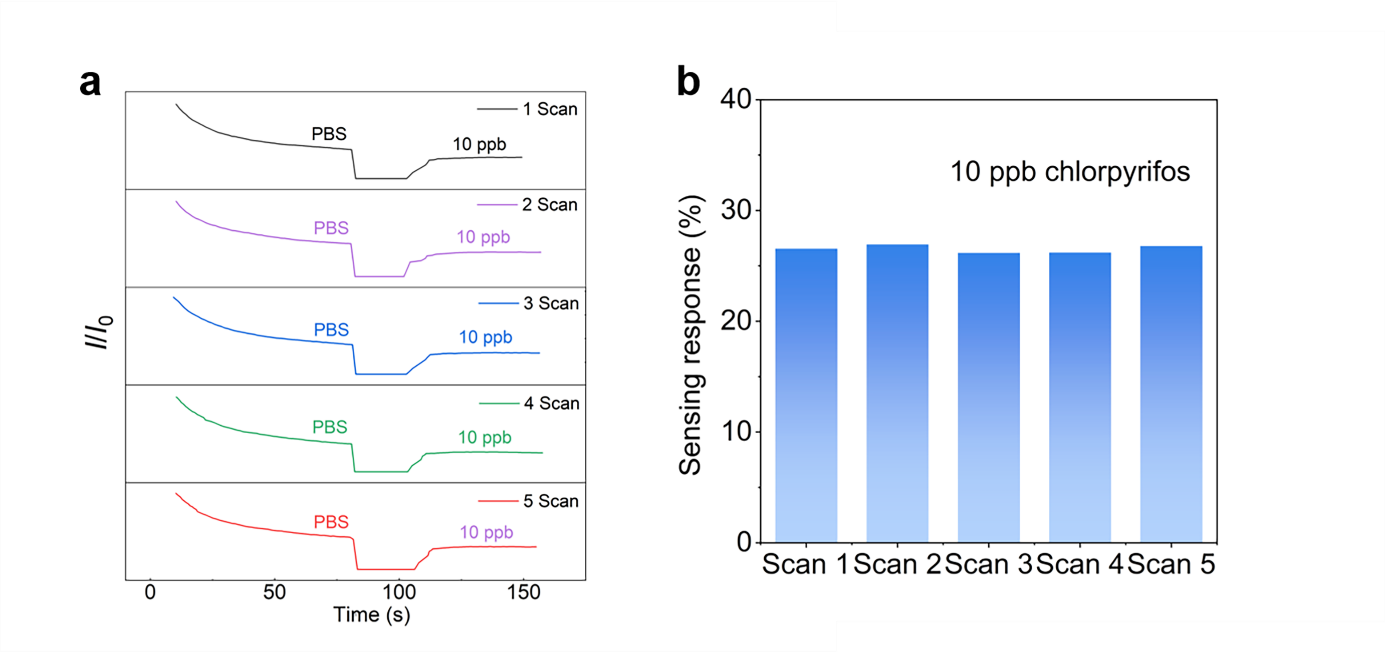


**Figure S24.** a) Normalized drain current (*I*/*I*_0_) toward 10 ppb chlorpyrifos for five consecutive tests on a single sensor. b) The sensing response values extracted from five consecutive scanning tests.


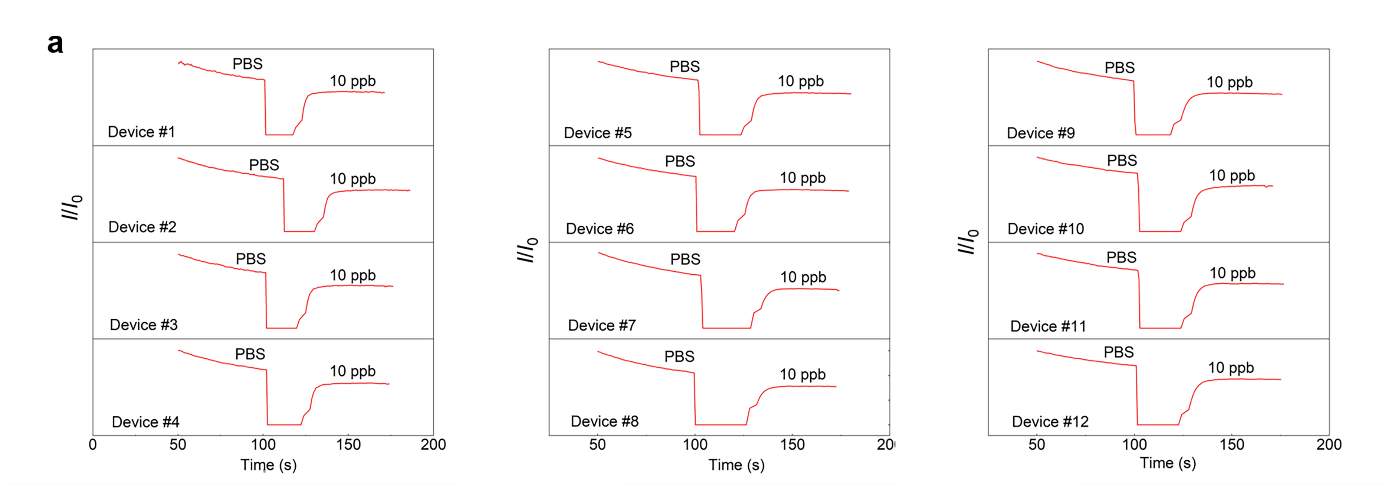


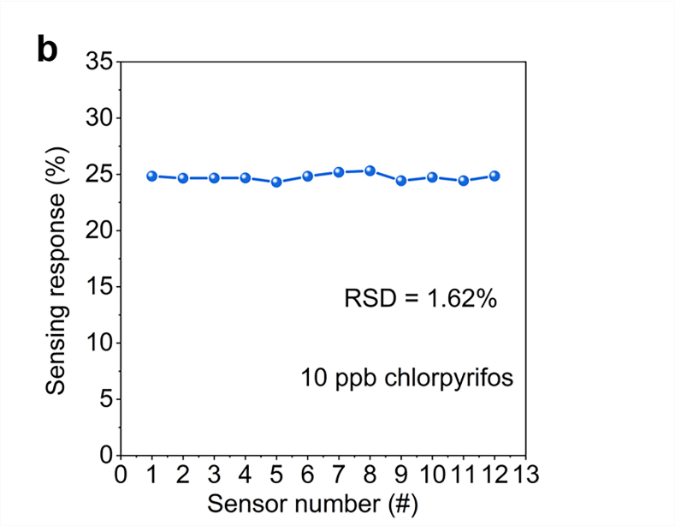


**Figure S25.** a) Normalized drain current (*I*/*I*_0_) toward 10 ppb chlorpyrifos of 12 OFET sensors. (b) The sensing response values extracted from 12 OFET sensors.


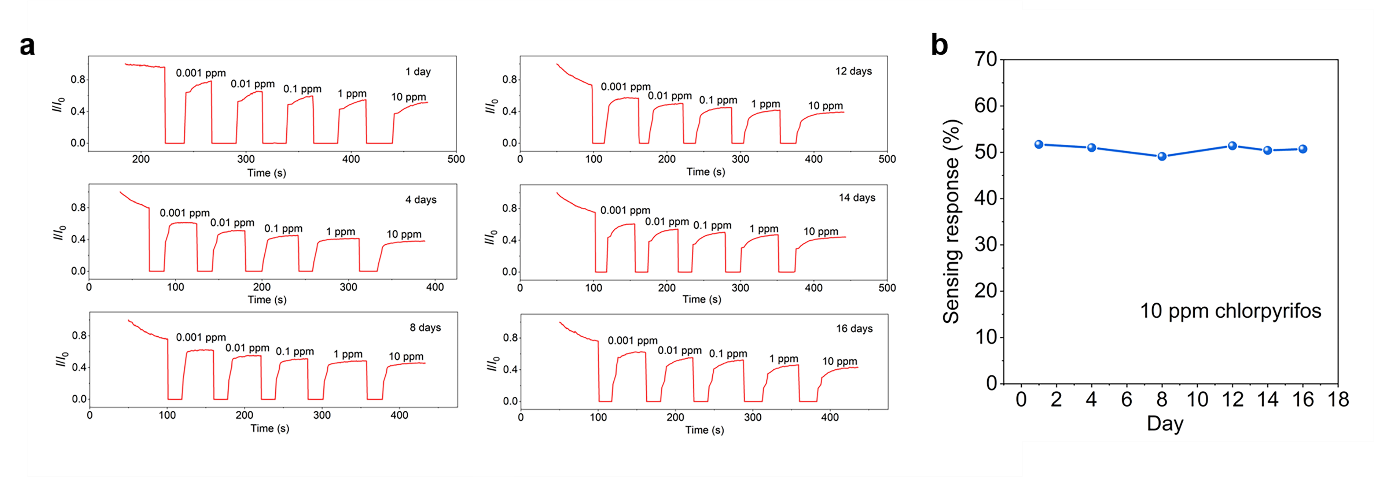


**Figure S26.** a) The OFET sensor consistently detected pesticides at concentrations ranging from 0.001 ppm to 10 ppm over 16 days. b) The sensing response stability of the OFET pesticide sensor.

**Supplementary Table 3.** Comparison of reported state-of-the-art pesticide sensors on response/recovery time, and long-term stability.


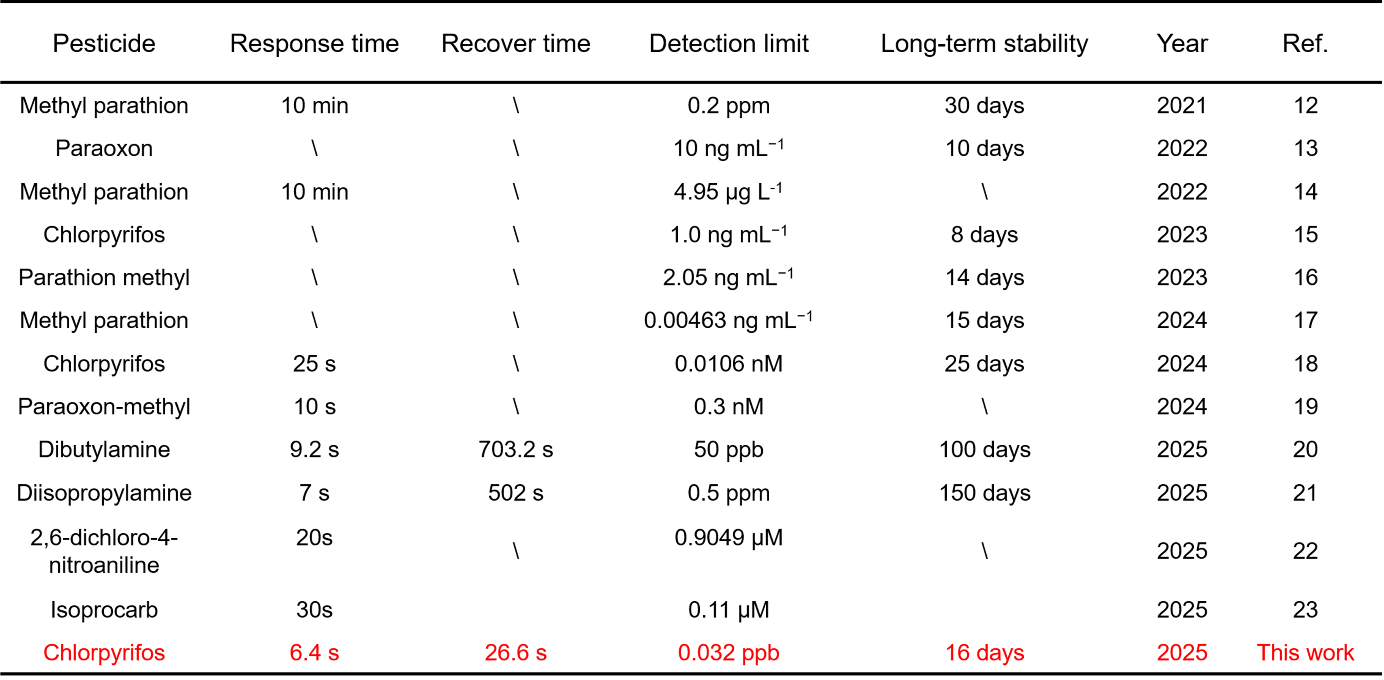


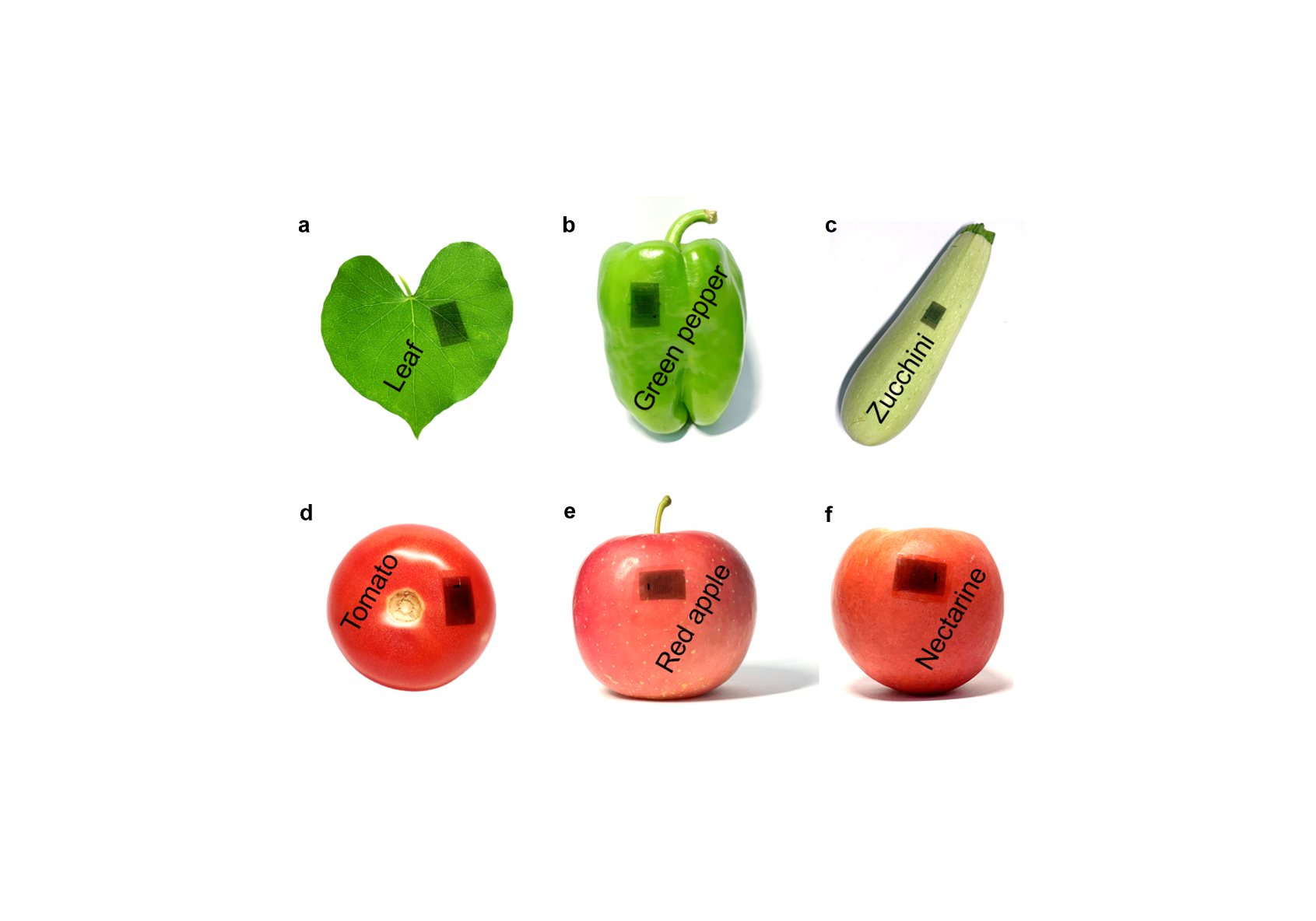


**Figure S27.** a-f) Photograph of the fully stretchable pesticide sensors placed on the leaf (a), green pepper (b), zucchini (c), tomato (d), red apple (e), and nectarine (f).

**Reference**

1. J. Xu, H.-C. Wu, C. Zhu, A. Ehrlich, L. Shaw, M. Nikolka, S. Wang, F. Molina-Lopez, X. Gu, S. Luo, D. Zhou, Y.-H. Kim, G.-J. N. Wang, K. Gu, V. R. Feig, S. Chen, Y. Kim, T. Katsumata, Y.-Q. Zheng, H. Yan, J. W. Chung, J. Lopez, B. Murmann, Z. Bao, Multi-scale Ordering in Highly Stretchable Polymer Semiconducting Films. *Nat. Mater.* **2019**, *18*, 594˗601.
2. J. Mun, J. Kang, Y. Zheng, S. Luo, H.-C. Wu, N. Matsuhisa, J. Xu, G.-J. N. Wang, Y. Yun, J. B.-H. Tok, Z. Bao, Conjugated Carbon Cyclic Nanorings as Additives for Intrinsically Stretchable Semiconducting Polymers. *Adv. Mater.* **2019**, *31*, 1903912.
3. Y. Zheng, G.-J. N. Wang, J. Kang, M. Nikolka, H.-C. Wu, H. Tran, S. Zhang, H. Yan, H. Chen, P. Y. Yuen, J. Mun, R. H. Dauskardt, I. McCulloch, J. B.-H. Tok, X. Gu, Z. Bao, An Intrinsically Stretchable High-performance Polymer Semiconductor with Low Crystallinity. *Adv. Funct. Mater.* **2019**, *29*, 1905340.
4. H. Ren, J. Zhang, Y. Tong, J. Zhang, X. Zhao, N. Cui, Y. Li, X. Ye, Q. Tang, Y. Liu, Synchronously Improved Stretchability and Mobility by Tuning the Molecular Weight for Intrinsically Stretchable Transistors. *J. Mater. Chem. C.* **2020**, *8*, 15646˗15654.
5. J. Mun, J. Kang, Y. Zheng, S. Luo, Y. Wu, H. Gong, J. Lai, H.-C. Eu, G. Xue, J. B.-H. Tok, Z. Bao, F4-TCNQ as an Additive to Impart Stretchable Semiconductors with High Mobility and Stability. *Adv. Electron. Mater.* **2020**, *6*, 2000251.
6. S. Wang, X. Zhao, C. Zhang, Y. Yang, J. Liang, Y. Ni, M. Zhang, J. Li, X. Ye, J. Zhang, Y. Tong, Q. Tang, Y. Liu, Suppressing Interface Strain for Eliminating Double-Slope Behaviors: Towards Ideal Conformable Polymer Field-Effect Transistors. *Adv. Mater.* **2021**, *33*, 2101633.
7. H.-W. Cheng, S. Zhang, L. Michalek, X. Ji, S. Luo, C. B. Cooper, H. Gong, S. Nikzad, J. A. Chiong, Y. Wu, Y. Zheng, Q. Liu, D. Zhong, Y. Lei, Y. Tomo, K.-H. Wei, D. Zhou, J. B.-H. Tok, Z. Bao, Realizing Intrinsically Stretchable Semiconducting Polymer Films by Nontoxic Additives. *ACS Materials Lett.* **2022**, *4*, 2328˗2336.
8. M. H. Kim, M. W. Jeong, J. S. Kim, T. U. Nam, N. T. P. Vo, L. Jin, T. I. Lee, J. Y. Oh, Mechanically Robust Stretchable Semiconductor Metallization for Skin-inspired Organic Transistors. *Sci. Adv.* **2022**, *8*, eade2988.
9. F. Wu, Y. Liu, J. Zhang, X. Li, H. Yang, W. Hu, Highly Stretchable and High-mobility Semiconducting Nanofibrous Blend Films for Fully Stretchable Organic Transistors. *Sci. China Mater.* **2023**, *66*, 1891˗1898.
10. J. Sun, X. Liu, Y. Tong, G. Zhao, Y. Ni, X. Zhao, B. Wang, X. Wang, M. Zhang, S. Guo, X. Han, Q. Tang, Y. Liu, Air/Liquid Interfacial Self-assembled Intrinsically Stretchable IDT-BT Film Combining a Deliberate Transfer Adherence Strategy for Stretchable Electronics. *ACS Appl. Mater. Interfaces* **2023**, *15*, 46108˗46118.
11. X. Li, A. Sabir, X. Zhang, H. Jiang, W. Wang, X. Zheng, H. Yang, Highly Stretchable and Oriented Wafer-Scale Semiconductor Films for Organic Phototransistor Arrays. *ACS Appl. Mater. Interfaces* **2024**, *16*, 36678˗36687.
12. A. M. Ulloa, N. Glassmaker, M. R. Oduncu, P. Xu, A. Wei, M. Cakmak, L. Stanciu, Roll-to-Roll Manufactured Sensors for Nitroaromatic Organophosphorus Pesticides Detection. *ACS Appl. Mater. Interfaces.* **2021,** *13*, 35961-35971.
13. H. Li, C. Su, N. Liu, T. Lv, C. Yang, Q. Lu, C. Sun, X. Yan, Carbon Dot-anchored Cobalt Oxyhydroxide Composite-based Hydrogel Sensor for On-site Monitoring of Organophosphorus Pesticides. *ACS Appl. Mater. Interfaces.* **2022,** *14,* 53340-53347.
14. L. Zhang, Y. Sun, Z. Zhang, Y. Shen, Y. Li, T. Ma, Q. Zhang, Y. Ying, Y. Fu, Portable and Durable Sensor Based on Porous MOFs Hybrid Sponge for Fluorescent-visual Detection of Organophosphorus Pesticide. *Biosens. Bioelectron.* **2022,** *216*, 114659.
15. M. Wu, Y. Du, H. Xu, X. Zhang, J. Ma, A. Li, L. Chou, Enzyme-engineered Metal-organic Frameworks for the Construction of Organophosphorus Pesticide Biosensor. *Adv. Funct. Mater*. **2024**, *34*, 2309383.
16. X. Wang, H. Yu, Q. Li, Y. Tian, X. Gao, W. Zhang, Z. Sun, Y. Mou, X. Sun, Y. Guo, F. Li, Development of a Fluorescent Sensor Based on TPE-Fc and GSH-AuNCs for the Detection of Organophosphorus Pesticide Residues in Vegetables. *Food Chem.* **2024**, *431*, 137067.
17. Y. Liu, Y. Xiao, Y. Zhang, X. Gao, H. Wang, B. Niu, W. Li, ZnO-rGO-based Electrochemical Biosensor for the Detection of Organophosphorus Pesticides. *Bioelectrochemistry*, **2024,** *156*, 108599.
18. S. Sakhtawat, S. K. Yadav, K. R.B. Singh, D. Kumar, J. Singh, Nanostructured Cadmium Sulfide-modified Screen-printed Carbon Electrode Based Electrochemical Sensor for Highly Efficient Quantification of Chlorpyrifos Pesticide in Water and Soil Samples. *J. Mol. Liq.* **2024**, *402*, 124711.
19. A. Sarma, S. Ghosh, S. Biswas, An Aluminium–organic Framework Unveiling Ultrasensitive Fluorometric Detection of Pesticide Paraoxon-methyl and Pharmaceutical Drug Azathioprine in Fruits, Vegetables, and Wastewater. *J. Mater. Chem. C*. **2024**, *12*, 13892-13903.
20. B. Li, Y. Liang, R. Liao, X. Zhang, M. Yuan, J. Zhang, D. Zhang, Y. Xu, In situ deposition of Bimetallic MgFe_2_O_4_ Nanotube Arrays Enables ppb-level Detection of Dibutylamine. *Appl. Surf. Sci.* **2025,** *707*, 163625.
21. M. Zhu, G. Chen, K. Song, J. Xin, X. Huang, Dual-selectivity and High-sensitivity Sensor for Dibutylamine and Diisopropylamine Detection Using ZnO Nanoparticles with Tetragonal Dipyramidal Morphology. *Appl. Surf. Sci.* **2025**, *701*, 163322.
22. W. Ding, X. Liu, W. A. Khokhar, C. Wei, F. U. Rehman, G. Wang, Z. Mahmood, A. R. Aleem, Rapid Fluorescent Sensing through Heparin and Chitosan Encapsulated Eu^3+^ Complexes for Pesticide Detection. *Int. J. Biol. Macromol.* **2025**, *308*, 142269.
23. H. Luo, L. Lai, C. Yu, Z. Zhou, C. Zeng, S. Sheng, Controlled Synthesis of Lanthanide Cluster and Coordination Polymer and as Sensors for Pesticide and Antibiotics. *Microchem. J.* **2025**, *210*, 112925.
